# Deconstructing the traditional Japanese medicine “Kampo”: compounds, metabolites and pharmacological profile of maoto, a remedy for flu-like symptoms

**DOI:** 10.1038/s41540-017-0032-1

**Published:** 2017-10-24

**Authors:** Akinori Nishi, Katsuya Ohbuchi, Hirotaka Kushida, Takashi Matsumoto, Keiko Lee, Haruo Kuroki, Shigeki Nabeshima, Chika Shimobori, Nagisa Komokata, Hitomi Kanno, Naoko Tsuchiya, Makoto Zushi, Tomohisa Hattori, Masahiro Yamamoto, Yoshio Kase, Yukiko Matsuoka, Hiroaki Kitano

**Affiliations:** 1 Tsumura Research Laboratories, Tsumura & CO., Ibaraki, Japan; 2 Comprehensive Kampo Research Planning Department, Tsumura & CO., Tokyo, Japan; 3 Sotobo Children’s Clinic, Medical Corporation Shigyo-no-kai, Chiba, Japan; 4 General Medicine, Fukuoka University Hospital, Fukuoka, Japan; 5 Product and General Administration Department, Tsumura & CO., Tokyo, Japan; 6 The Systems Biology Institute, Tokyo, Japan; 7 Laboratory for Disease Systems Modeling, RIKEN Center for Integrative Medical Sciences, Kanagawa, Japan; 8 Okinawa Institute of Science and Technology, Okinawa, Japan; 9 Sony Computer Science Laboratories, Inc, Tokyo, Japan

## Abstract

Pharmacological activities of the traditional Japanese herbal medicine (Kampo) are putatively mediated by complex interactions between multiple herbal compounds and host factors, which are difficult to characterize via the reductive approach of purifying major bioactive compounds and elucidating their mechanisms by conventional pharmacology. Here, we performed comprehensive compound, pharmacological and metabolomic analyses of maoto, a pharmaceutical-grade Kampo prescribed for flu-like symptoms, in normal and polyI:C-injected rats, the latter suffering from acute inflammation via Toll-like receptor 3 activation. In total, 352 chemical composition-determined compounds (CCDs) were detected in maoto extract by mass spectrometric analysis. After maoto treatment, 113 CCDs were newly detected in rat plasma. Of these CCDs, 19 were present in maoto extract, while 94 were presumed to be metabolites generated from maoto compounds or endogenous substances such as phospholipids. At the phenotypic level, maoto ameliorated the polyI:C-induced decrease in locomotor activity and body weight; however, body weight was not affected by individual maoto components in isolation. In accordance with symptom relief, maoto suppressed TNF-α and IL-1β, increased IL-10, and altered endogenous metabolites related to sympathetic activation and energy expenditure. Furthermore, maoto decreased inflammatory prostaglandins and leukotrienes, and increased anti-inflammatory eicosapentaenoic acid and hydroxyl-eicosapentaenoic acids, suggesting that it has differential effects on eicosanoid metabolic pathways involving cyclooxygenases, lipoxygenases and cytochrome P450s. Collectively, these data indicate that extensive profiling of compounds, metabolites and pharmacological phenotypes is essential for elucidating the mechanisms of herbal medicines, whose vast array of constituents induce a wide range of changes in xenobiotic and endogenous metabolism.

## Systems biology of traditional medicine: comprehensive analysis of a traditional Japanese medicine, maoto

Pharmacological activities of Kampo, or traditional Japanese herbal medicine, are putatively mediated by complex interactions between the plant-derived compounds and endogenous molecules. To elucidate these properties, we performed comprehensive phytochemical profiling, and pharmacological and metabolomic analyses of maoto, an herbal remedy for flu-like symptoms. In the plasma of maoto-treated rats, we detected maoto-derived compounds, metabolites produced from the chemical transformation of maoto compounds by host metabolism and gut microbes, and endogenous metabolites that were appeared following maoto administration. In an acute inflammatory rat model, maoto ameliorated symptoms of sickness behavior, suppressed inflammatory cytokines, and extensively affected common metabolites and lipid mediators. These data suggest that the diverse chemical composition of Kampo broadly affects the host’s endogenous metabolism and exerts specific pharmacological effects.

## Introduction

Traditional herbal medicine (THM) is widely accepted and used throughout the world, especially in Asia. Although there is diversity among THMs, such as traditional Chinese medicine, Tibetan medicine, Indian Ayurveda, and Japanese Kampo medicine, they are empirically formulated and generally acknowledged to work for certain cases.

Among various herbal remedies in the world, Kampo is unique as a therapeutic agent that has been modernized through technological and manufacturing innovation. Furthermore, Kampo is covered by the national health insurance scheme and is manufactured in accordance with both good manufacturing practice (GMP) stipulated by Japanese law and Kampo GMP guidelines^1^ to ensure stringent quality control at all stages of production—a key element that is pivotal for the reproducibility and standardization of biological effects, as well as for rigorous scientific investigation.

Maoto, a pharmaceutical grade Kampo (TSUMURA Maoto Extract Granules, TJ-27; Ma Huang Tang in Chinese) prepared from a mixture of four component herbs, Armeniacae semen (AS) (32.3%), Glycyrrhizae radix (GR) (9.6%), Cinnamomi cortex (CC) (25.8%), and Ephedrae herba (EH) (32.3%), is prescribed widely to treat symptoms of upper respiratory infections and influenza. Clinical studies have shown that maoto has antipyretic^2,3^ and anti-malaise effects^4^ in children, and improves flu symptoms with efficacy comparable to that of neuraminidase inhibitors in adults infected with influenza A virus.^5,6^ Experimentally, maoto has been shown to decrease viral titer, while exerting an antipyretic effect.^7^ Furthermore, the herbal compounds in maoto have been reported to have various modulating effects on the host’s immune system, including Toll-like receptors (TLR)^8–14^ and a direct inhibitory effect on viral growth.^15–19^ However, the mechanism of action of maoto in acute infectious disorders is largely unclarified due to the presence of large and diverse compounds.

Many THMs are considered to function through the synergetic effects of the combined herbs; in other words, multiple active compounds exert multifaceted influences on the body. Understanding which compounds are involved in this process and how they give rise to synergetic effects are two major questions that must be answered to understand THM.

To approach these questions, we need to address the complex features of THM, step by step, with newly developed methodologies suitable for analyzing the multiple effects of such medicines. As a first step, it is important to characterize comprehensively the compound profile in the body after treatment with THM, mainly by mass spectrometric analyses. Notably in THM, many compounds present in the raw herbs are metabolized and converted by the host xenobiotic system and gut microbial flora. In order to identify the actual compounds involved in THM efficacy, it is necessary to identify compounds that are present in the blood after THM administration, as well as those present in the herbal extract. Therefore, exhaustive measurements of THM-derived compounds in both extracts and plasma are necessary. Furthermore, because THM contains numerous bioactive compounds that are thought to have a deep and wide-ranging impact on host metabolism, THM-induced changes in host metabolites are likely to exert profound influences on human health and disease. Metabolomic analysis on tissue samples of THM-treated humans, both healthy and diseased, is therefore is indispensable in understanding the mode of action of THM, which is essentially different from that of Western medicines based on a single compound.

In the present study, we have taken an integrated approach of pharmacokinetics (PK) and metabolomics to investigate the effect of the Japanese Kampo maoto on host immune response in acute viral infection. We used a rat model of immune activation induced by a viral mimic, polyinosinic–polycytidylic acid (poly I:C), which is an agonist of TLR3 and causes fever, fatigue (decreased locomotor activity), reduced food intake, body weight loss and inflammatory cytokine surge, all of which reflect systemic symptoms or phenotypes in patients with influenza-like illness.^20,21^ Using this model, we performed metabolomic analyses of both maoto drug extract and plasma after maoto administration in normal and diseased rats, and evaluated the pharmacological effects on disease symptoms, accompanied by an authentic PK study. This approach has enabled comprehensive profiling of the changes in maoto-derived compounds, endogenous metabolites, and inflammatory/symptomatic responses in the host, providing an integrated picture of the biological impact of maoto on the body.

## Results

**Fig. 1:**
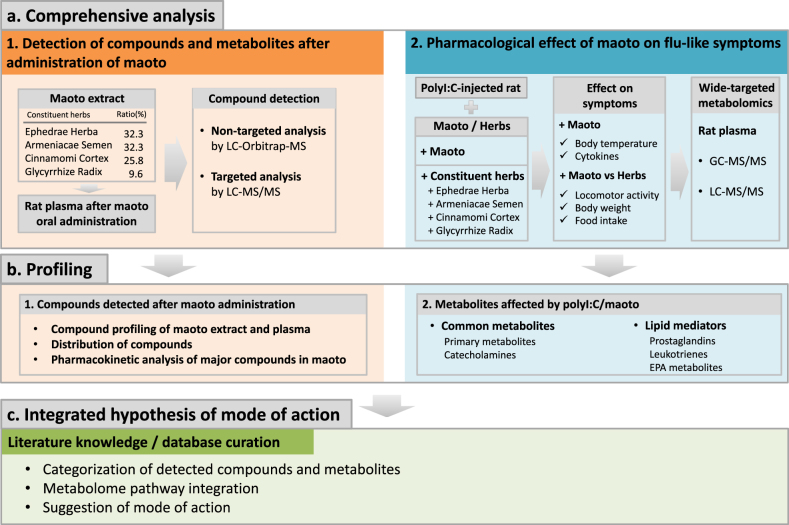
Schematic view of the workflow in this study. **a1** Pharmacokinetics analysis of major compounds was performed. Original compounds in maoto, and their metabolites in plasma after maoto administration were measured by non-targeted and targeted analysis. **a2** Pharmacological analysis of the effect of maoto on flu-like symptoms was performed by conventional pharmacology and targeted metabolome analysis. **b1** MS peaks were annotated to determine the compounds, and the data for compounds originating from maoto, and endogenous metabolites affected by maoto administration were summarized. **b2** Common metabolites and lipid mediators affected by maoto administration were summarized. **c** To integrate the obtained data, the detected compounds and metabolites were categorized and pathway maps were built and annotated by literature and database knowledge curation

The workflow of this study is presented in Fig. 1. In a series of analyses, maoto extract and rat plasma after maoto administration were analyzed by liquid chromatography–Orbitrap-mass spectrometry (LC-Orbitrap-MS) to compare the differences in compound profile. The PK parameters of major compounds in maoto were measured by LC-MS/MS in plasma after maoto administration to rat (Supplementary Table 1). The effects of maoto on polyI:C-induced flu-like symptoms, such as decreased locomotor activity and body weight, and inflammatory cytokine response were evaluated. Metabolomic changes in primary metabolites and lipid mediators were analyzed in rat plasma by gas chromatography (GC)-MS/MS and LC-MS/MS after maoto and polyI:C treatment. The compounds and metabolites detected in these analyses were categorized and built into pathway maps, and their functions were annotated by literature/database curation. Lastly, the integrated analysis enabled us to summarize the mode of action of maoto. The summarized data are shown in Supplementary Fig. 1.

### Compound and metabolite profiling by LC-Orbitrap-MS analysis after administration of maoto

**Fig. 2:**
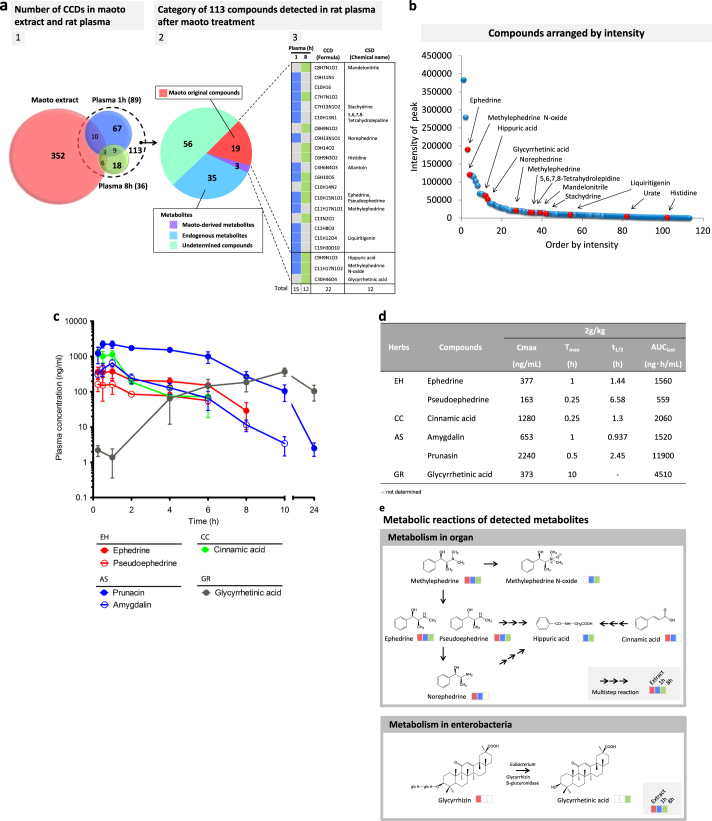
Profiles of compounds in maoto extract, and compound/metabolites detected in rat plasma after maoto administration. **a1** Profiles of compounds in maoto extract and those that specifically appeared/increased in rat plasma at 1 h and 8 h after maoto administration (2 g/kg). The number of chemical composition-determined (CCD) compounds detected by non-targeted analysis in maoto extract (salmon pink), and specifically in rat plasma at 1 h (blue) and 8 h (light green) after maoto administration are shown in the Venn diagram. **a2** Of the 113 CCD compounds detected in plasma, the proportions of original maoto compounds (salmon pink) and metabolites subcategorized as maoto-derived metabolites (purple), endogenous metabolites (light blue), and undetermined compounds (mint green) are shown as a pie chart. **a3** The chemical formulae and names of original maoto compounds and maoto-derived compounds are shown as color-coded graphs. **b** Distribution of CCD compounds specifically detected in rat plasma by non-targeted analysis after maoto administration. The CCD compounds are arranged in descending order from high to low peak intensity at 1 h and 8 h after maoto treatment. If the same CCD compounds were detected at 1 h and 8 h after treatment, the higher intensity was used. **c** Targeted LC-MS/MS analysis of the PK properties of major maoto compounds in plasma after maoto administration (2 g/kg). Among 21 major compounds that were measured, the PK properties of compounds with relatively high concentrations in plasma are shown. The compounds derived from Ephedrae Herba (EH), Armeniacae Semen (AS), Cinnamomi Cortex (CC) and Glycyrrhizae Radix (GR) are marked in red, blue, green and gray, respectively. Data represent mean ± SD (*n* = 3 for each time point). **d** Plasma PK parameters of six major maoto compounds in plasma after oral administration of maoto (2 g/kg). **e** Major metabolic reactions of original compounds in maoto detected by non-targeted analysis. Compounds detected in maoto extract and rat plasma at 1 h and 8 h after maoto administration (2 g/kg) are shown as color-coded graphs (salmon pink, light blue, and mint green, respectively). A single arrow indicates a compound metabolized in a single reaction; a series of three arrows indicates a compound metabolized in a multistep reaction. Ephedrines (methylephedrine, ephedrine and pseudoephedrine) are metabolized to methylephedrine *N*-oxide or hippuric acid, and cinnamic acid is metabolized to hippuric acid in organs such as the liver in vivo. Glycyrrhizin, a glycoside, is metabolized to an aglycon (glycyrrhetinic acid) by glucuronidase of Enterobacteria

LC-Orbitrap-MS analysis of compounds and metabolites in maoto extract and in rat plasma at 1 and 8 h after oral administration was performed. The combined results are summarized in Fig. 2a. In total, 113 composition-determined compounds (CCDs) were newly detected in rat plasma after maoto treatment. Of these, 19 were present in maoto extract, corresponding to 5.4% of the 352 CCDs originally present in maoto extract, while the other 94 CCDs were presumed to be metabolites generated from either maoto compounds or endogenous substances because they were not detected in maoto extract or control plasma.

The data from the LC-Orbitrap-MS analysis of maoto extract and rat plasma after oral administration, including the percentage of detected compounds, CCDs and chemical structure-determined compounds, are summarized in Supplementary Table 2. The chemical structure of only a small proportion of the CCDs, i.e., 88 of 352 CCDs in maoto extract and 22 of 89 CCDs in the rat plasma at 1 h after administration (Supplementary Table 2), were identified by using existing chemical databases.

Among the 113 compounds detected in rat plasma after maoto administration, 19 were found in maoto extract (e.g., ephedrine, methylephedrine, norephedrine, liquiritigenin, 5,6,7,8-tetrahydrolepidine, mandelonitrile, etc.) and 3 were maoto-derived metabolites generated by rat and/or its enteric flora (methylephedrine *N*-oxide, hippuric acid and glycyrrhetinic acid). A further 35 CCDs were host-derived endogenous metabolites (phospholipids and acylcarnitines), whereas the source of the remaining 56 CCDs could not be determined (Fig. 2a). The details of 35 endogenous metabolites (28 phospholipids, 4 acylcarnitines, and 3 metabolites) and the undetermined 56 CCDs are listed in Supplementary Fig. 2. The arrangement of compounds detected by non-targeted analysis in descending order from high to low peak intensity showed a statistical long-tail distribution (Fig. 2b).

### Plasma PK profiling of the major compounds of maoto

To supplement the non-targeted analysis, we performed a targeted PK analysis of well-curated maoto compounds identified through a literature search. We used the LC-MS/MS method and simultaneously analyzed the PK profiles of 21 major compounds of maoto after oral administration in rats (Supplementary Table 1). It should be noted that, due to methodological limitations, some well-known maoto compounds such as amygdalin, prunasin, and cinnamic acid were not detected by non-targeted analysis in this study (see Supplementary Methods). Of the 21 compounds, 19 were detected in the plasma of rats given maoto (2 g/kg) (Supplementary Fig. 3), and 6 showed a relatively higher concentration in plasma than the other compounds (Fig. 2c, d). In terms of PK profile, the following five compounds reached relatively high concentrations within 1 h of administration (*T*_max_: 0.25–1.00 h), and were eliminated comparably from blood (t_1/2_: 0.937 ~ 6.58 h): ephedrines (ephedrine and pseudoephedrine) from EH; amygdalin and its metabolite prunasin from AS; and cinnamic acid, a metabolite of cinnamaldehyde from CC. The maximum concentration of these compounds (*C*_max_) was 377, 163, 653, 2240, and 1280 ng/mL, respectively (Fig. 2c, d). On the other hand, the concentration of glycyrrhetinic acid, an aglycon of glycyrrhizin, which is a major compound in GR, gradually increased and reached *C*_max_ (373 ng/mL) 10 h after administration (Fig. 2c, d; see Supplementary Fig. 3 for the profiles of the 19 major compounds). The known metabolic pathways of the major compounds detected are shown in Fig. 2e and Supplementary Fig. 4.

### Pharmacological profiling of maoto

**Fig. 3:**
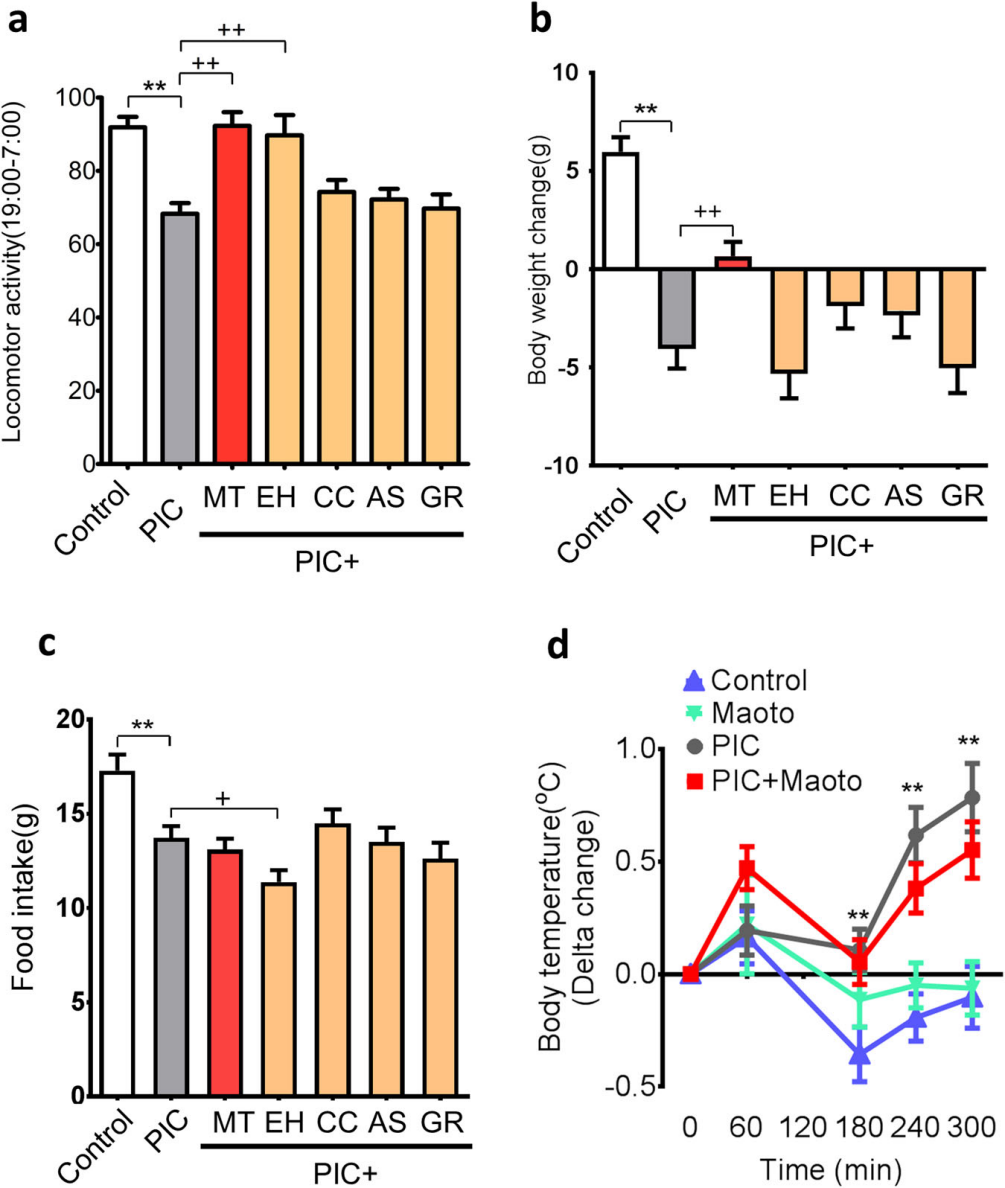
Effects of maoto and its constituent herbs on polyI:C (PIC)-induced sickness phenotypes. **a** Percentage change in locomotor activity, **b** changes in body weight, **c** changes in food intake, and **d** body temperature. The change in each group is relative to the value measured immediately before maoto (MT) administration. PolyI:C was administered at a dose of 6 mg/kg. Maoto was administered at a dose of 2 g/kg, and its constituent herbs (Ephedrae Herba (EH), Armeniacae Semen (AS), Cinnamomi Cortex (CC), and Glycyrrhizae Radix (GR)) were administered at doses equivalent to their respective content in 2 g/kg of maoto (EH, 645 mg/kg; AS, 645 mg/kg; CC, 516 mg/kg; GR, 194 mg/kg). Data represent mean ± SEM of the following measurements: **a**, **b**, and **c**, Control (*n* = 19), PIC (*n* = 23), PIC + MT (*n* = 23), PIC + EH (*n* = 18), PIC + CC (*n* = 23), PIC + AS (19) and PIC + GR (19); **d**, *n* = 10. **P < 0.01 versus control group; ^++^ *P* < 0.01, ^+^ *P* < 0.05 versus polyI:C group by Welch’s *t*-test with Bonferroni correction

We analyzed the effect of maoto and its constituent herbs on the symptoms induced by administration of polyI:C, which causes certain flu-like symptoms, especially fever and fatigue in rats. Administration of maoto (2 g/kg) suppressed the decrease in locomotor activity and body weight induced by polyI:C injection (Fig. 3a, b); however, it did not significantly affect the reduced food intake (Fig. 3c) or febrile response (Fig. 3d). On the other hand, maoto administration in normal rats increased locomotor activity and decreased food intake, but did not significantly affect body weight (Supplementary Fig. 5a–c).

A similar improvement in locomotor activity was observed after administration of EH (Fig. 3a). Unlike maoto, however, administration of extract from the individual herbs did not ameliorate the body weight loss induced by polyI:C (Fig. 3b); and maoto analog, comprising a mixture of three constituent herbs in maoto without HE (AS, CC and GR; designated EH(−)-maoto) did not affect the polyI:C-induced body weight loss and the decrease in locomotor activity (Supplementary Fig. 6). These results indicated that neither EH nor EH(−)-maoto could improve the body weight loss due to polyI:C, suggesting that a combination of multiple herbs is essential for this effect.

**Fig. 4:**
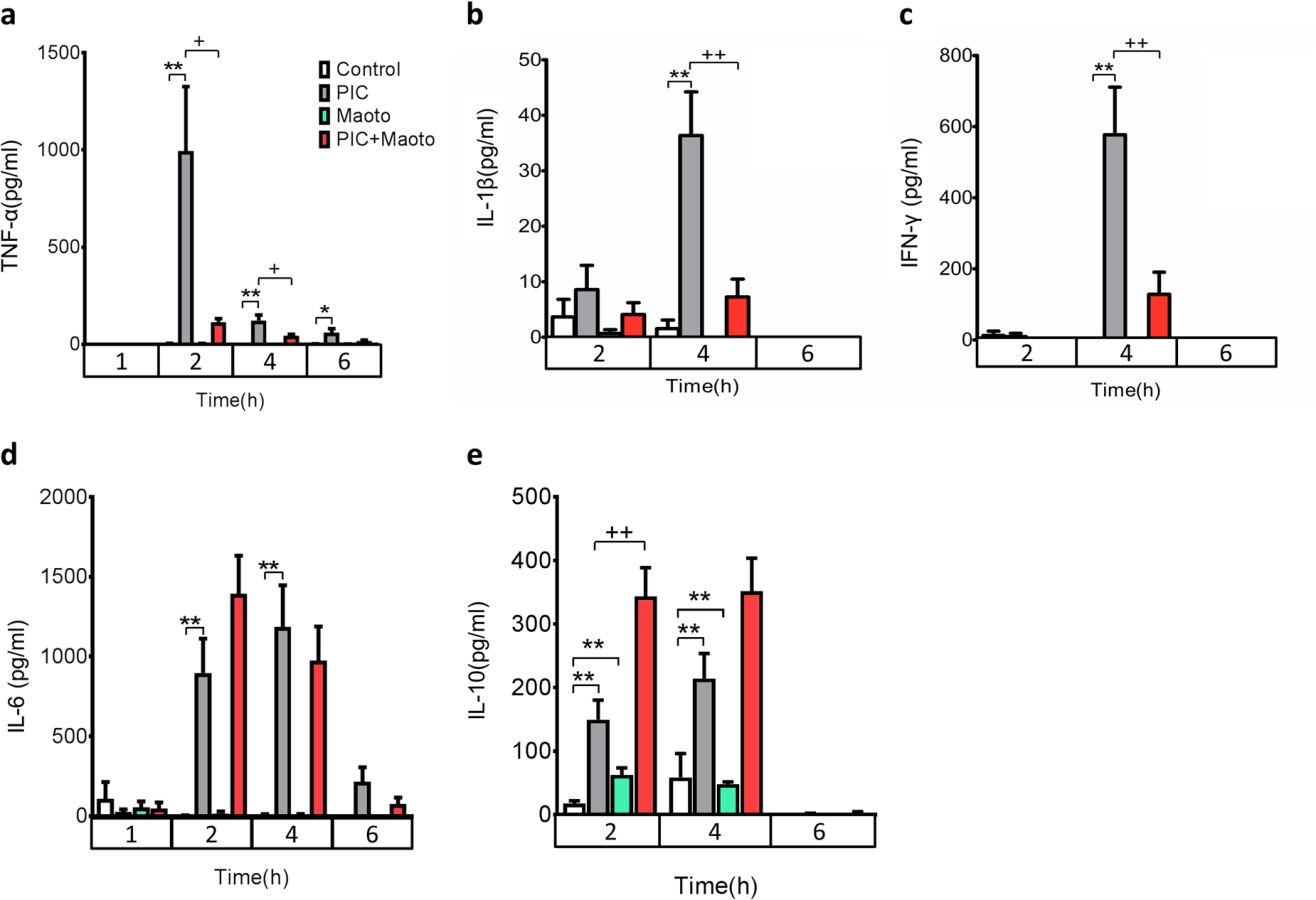
Effects of maoto on polyI:C (PIC)-induced cytokine response. Effect of maoto on polyI:C-induced cytokine levels: **a** TNF-α, **b** IL-1β, **c** IFN-γ, **d** IL-6, and **e** IL-10. PolyI:C was administered at a dose of 6 mg/kg. Maoto was administered at a dose of 2 g/kg. Relative plasma concentrations of cytokines at 1, 2, 4, and 6 h after maoto administration were measured. Data represent mean ± SEM of the following measurements: at 1 h, *n* = 6; at 2 h, Control (*n* = 18), PIC (*n* = 18), Maoto (*n* = 17), PIC + Maoto (*n*-18); at 4 h, TNF-α (*n* = 10); IL-1β (*n* = 10), IL-10 (*n* = 10), and IFN-γ (*n* = 10)**;** at 4 h, Control (*n* = 9), PIC (*n* = 10), Maoto (*n* = 10), PIC + Maoto (*n* = 10); IL-6 (*n* = 10); at 6 h, *n* = 6. **P < 0.01; *P < 0.05 versus control group; ^++^ *P* < 0.01, ^+^ *P* < 0.05 versus polyI:C group by Welch’s *t*-test with Bonferroni correction

Among the cytokines upregulated by polyI:C, administration of maoto (2 g/kg) significantly attenuated the induction of tumor necrosis factor (TNF)-α and interleukin (IL)-1β (Fig. 4a, b), as well as interferon (IFN)-γ (Fig. 4c), whereas IL-6 remained unaffected (Fig. 4d). By contrast, IL-10 induction was significantly enhanced (Fig. 4e). The effect of maoto on TNF-α and IL-10 was confirmed at lower doses of 0.25 and 0.5 g/kg (Supplementary Fig. 7a, b).

### Metabolomic profiling of maoto by GC-MS/MS

**Fig. 5:**
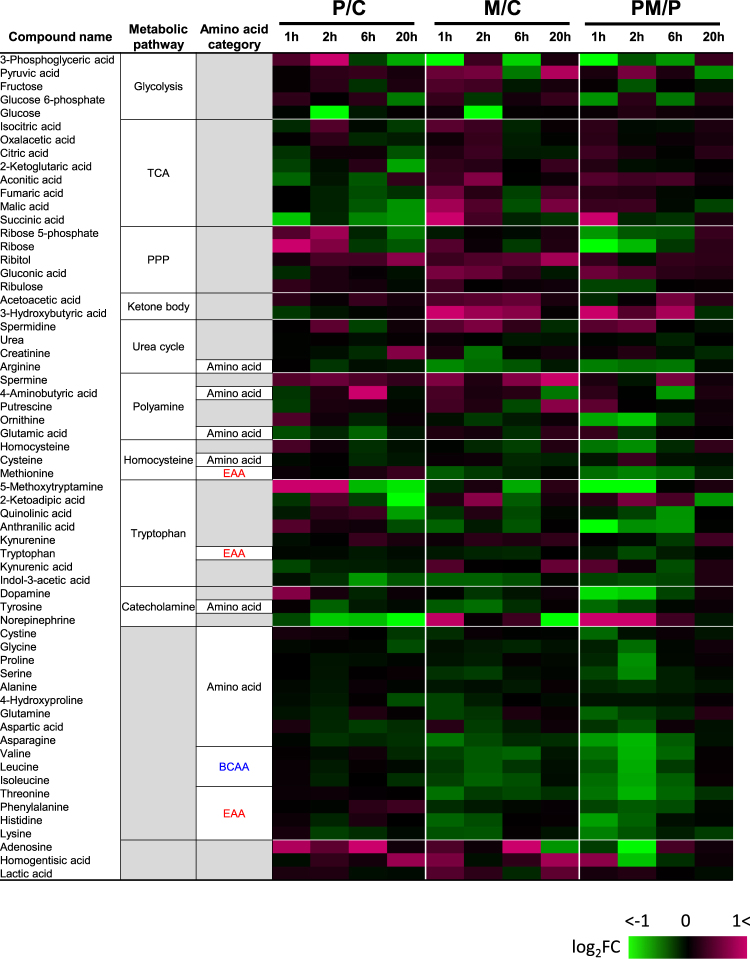
Heat map of changes in common metabolite levels in polyI:C-treated and/or maoto-treated rats. Heat map showing the fold change of each common metabolite in plasma after administration of polyI:C in control (P/C), maoto in control (M/C), and maoto in polyI:C-treated (PM/P) rats. Magenta indicates an increase in metabolite fold number or up-regulation; green indicates down-regulation of a specific metabolite versus the control (*n* = 6). *BCAA* branched-chain amino acid, *EAA* essential amino acid, *PPP* pentose phosphate pathway, *TCA* tricarboxylic acid

Next, we carried out metabolomic analysis after maoto administration by using GC-MS/MS as shown in Fig. 5 and Supplementary Table 3. The metabolites of tricarboxylic acid (TCA) cycle were downregulated and those of pentose phosphate pathway (PPP) were upregulated in the plasma of polyI:C-treated rats. In addition, polyI:C consistently decreased norepinephrine and increased adenosine.

Administration of maoto to polyI:C-treated or untreated rats increased ketone bodies. Furthermore, maoto treatment led to a decrease in amino acids, particularly those categorized as essential amino acids and branched-chain amino acids (BCAAs), in both normal and polyI:C-treated rats.

### Metabolomic profiling of maoto by LC-MS/MS

**Fig. 6:**
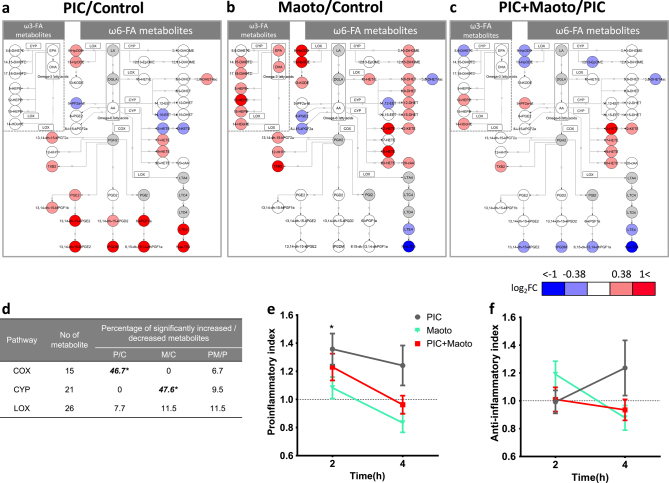
Profile of lipid mediators after administration of polyI:C and/or maoto. Comparison of fold changes of lipid mediators in plasma after administration of **a** polyI:C in control (PIC/Control(P/C)), **b** maoto in control (Maoto/Control(M/C)), and **c** maoto in polyI:C-treated (PIC + Maoto/PIC(PM/P)) rats, shown as metabolic pathways of lipid mediators. These pathways indicate fatty acids and lipid mediators that increased or decreased by > 0.38 or <−0.38 log fold change in response to each treatment, as well as key metabolites in the pathways. Circles indicate metabolites; undetected metabolites are colored gray. Rectangles indicate enzymes (COX, CYP and LOX) involved in metabolizing the lipid mediators. EPA and DHA are categorized as ω3 fatty acids; LA, DGLA, and AA are categorized as ω6 fatty acids. **d** Summary of changes in the metabolic pathways of lipid mediators. Fisher’s exact test was used to assess the effects of each treatment on lipid mediators categorized by the COX, CYP and LOX pathways (*P < 0.05). All detected lipid mediators metabolized by the COX, LOX, and CYP pathways were included in the test, and metabolites that significantly decreased or increased against each control group (*P* < 0.05 by Welch’s *t*-test) were analyzed. The metabolites in each metabolic pathway that significantly increased or decreased are listed in Supplementary Information. **e** Proinflammatory index and **f** anti-inflammatory index calculated as described by Tam et al.^22^ with modification. Detected lipid mediators were categorized by proinflammatory or anti-inflammatory index as described in Supplementary Information. The score of each group was normalized to the score of its own control group. Data represent mean ± SEM of the following measurements: at 2 h, Control (*n* = 18), Maoto (*n* = 17), PIC (*n* = 17), PIC + Maoto (*n* = 17); at 4 h, *n* = 10. *P < 0.05 versus control group by Welch’s *t*-test with Bonferroni correction

Further metabolomic analysis of lipid mediators in the plasma 2 h after treatment was conducted using LC-MS/MS (Fig. 6, Supplementary Fig. 8 and Supplementary Table 4). The data showed that polyI:C administration in normal rats mainly led to the upregulation of metabolites of arachidonic acid (AA), such as prostaglandins (PGs) and leukotrienes (LTs) (Fig. 6a). By contrast, maoto administration in normal rats did not affect PGs, although it significantly decreased the metabolites of LTs, such as LTE4 and N-acetyl-LTE4 (Fig. 6b). Maoto also increased several ω6 fatty acid metabolites such as hydroxy-eicosatetraenoic acids (HETEs) and dihydroxy-eicosatetraenoic acids (DHETs), which are alternative metabolites generated from AA (Fig. 6b). Moreover, maoto increased other ω-6 metabolites, including dihydroxyoctadecenoic acids, which originate upstream of AA (Fig. 6b).

When maoto was administered to polyI:C-injected rats, the opposing effects of polyI:C and maoto on the metabolic fate of AA negated each other, resulting in no change in PGs and a moderate increase in HETEs (Fig. 6c). Furthermore, maoto treatment increased plasma ω3 fatty acids metabolites such as eicosapentaenoic acid (EPA) and docosahexaenoic acid (DHA), as well as their metabolites (Fig. 6b). Considering the metabolic enzymes associated with each metabolite, polyI:C seemed to increase AA metabolites that are generated by cyclooxygenases (COXs) and lipoxygenases (LOXs), whereas maoto increased metabolites generated by cytochrome P450s (CYPs), as shown in Fig. 6d.

We carried out a cluster analysis of fatty acid metabolites at 2 h and 4 h after administration of polyI:C and maoto (Supplementary Fig. 8). At 4 h after administration, many AA-derived eicosanoid metabolites also increased in polyI:C-treated rats, although the types of metabolites that were increased differed substantially as compared with 2 h. By contrast, maoto administration to both polyI:C-treated and untreated rats led to a decrease in almost all eicosanoid metabolites.

Lastly, to clarify the global changes in lipid metabolites, we grouped the bioactive lipids based on their proinflammatory or anti-inflammatory properties and calculated the proinflammatory and anti-inflammatory indices, as described by Tam et al.^22^ PolyI:C injection led to a higher proinflammatory index at 2 h. At 4 h, both the proinflammatory and anti-inflammatory indices were higher than in the other groups, although the differences were not statistically significant. Maoto treatment in all groups resulted in indices closer to 1, which represents the value of naïve rats (Fig. 6e, f).

## Discussion

In the present study, compound profiling using experimentally substantiated PK analyses and targeted/non-targeted metabolomics confirmed the presence of specific and well-known major compounds in maoto. Ephedrines (ephedrine and pseudoephedrine), amygdalin and its metabolite prunasin, cinnamic acid, a metabolite of cinnamaldehyde, and glycyrrhetinic acid, a metabolite of glycyrrhizin produced by gut microbiota,^23^ were detected in the plasma in appreciable amounts. Among these compounds, ephedrines are known for their sympathomimetic effect.^24,25^ Ephedrines, amygdalin, and particularly glycyrrhetinic acid, have also been reported to possess various anti-inflammatory effects.^9,13,26^ However, these constitute only a small fraction of the original compounds in the maoto extract. Most of the compounds in maoto were not detected in blood, at least in their original forms. On the other hand, a considerable number of maoto compounds were presumably converted to other forms by enterobacteria and the xenobiotic system in the intestines and liver in the host, before, during or after absorption, resulting in compositional alteration over time.^23^ In this study, we detected the compound profile only in blood collected from the abdominal inferior vena cava, which includes compounds degraded by liver. It is important to consider the metabolism of maoto compounds in the liver because it is involved in the excretion of maoto compounds as xenobiotics and in the conversion of maoto derived compounds to pharmacologically active compounds. Our future studies will include metabolome analysis, non-target analysis, and metabolism study of maoto components in the liver tissue to reveal the pharmacological active compounds and PK/PD interactions. To elucidate the absorption of maoto-derived compounds, it will be necessary to compare compound profiles among blood from the abdominal inferior vena cava, blood from the portal vein, and urine.

Non-targeted analysis is useful for studying compound profiles in biological samples, but it also has several challenges. In this study, for example, amygdalin was detected by targeted analysis but not non-targeted analysis. However, we manually identified a peak predicted to be amygdalin in the MS/MS spectrum that was returned as an unknown peak for which the composition formula could not be determined. It is likely that the composition formula could not be calculated because evaluation of the adduct ion was not successful. Improving the determination accuracy of compound peaks is a future task for non-targeted analysis.

Our study also showed that endogenous metabolites had been concomitantly produced by 1 h after maoto administration. Taken together, our results imply that (1) the initial process of conversion of herbal compounds induces metabolic perturbations within 1 h, which might contribute to the initial physiological changes induced by maoto; and (2) newly produced maoto-derived and endogenous metabolites might potentiate and/or modify subsequent pharmacological effects. Thus, a comprehensive elucidation of serial plasma profiles of THM-derived and endogenous metabolites after THM administration is essential for delineating the mechanisms of action of THM. To aid this approach, however, databases of metabolites, particularly metabolites of herbal compounds produced by human and enteric microbiota should be developed because here we could not determine the chemical structures of many metabolites that were detected in blood after maoto administration due to the lack of MS and MS/MS data for such metabolites.

In the present study, using a polyI:C injection model, we observed the beneficial effect of maoto on fatigue symptoms, as previously reported in clinical settings. The observed effects of maoto on cytokines and flu-like symptoms are in good agreement with previous animal and clinical studies indicating that fatigue symptoms are tightly regulated by proinflammatory cytokines, particularly IL-1β.^27–29^ Furthermore, such modulatory effects of maoto on proinflammatory and anti-inflammatory cytokines might contribute to the reported benefit of this medicine on other disorders such as fever, arthralgia/myalgia, asthma, and allergic symptoms in clinical settings.^7,30,31^ The effect of maoto on these cytokines may be partially explained by the increase in some of its major compounds/metabolites, such as ephedrines and EPA, in plasma after administration. These compounds have been reported to promote IL-10 secretion and inhibit proinflammatory cytokines,^13,32^ although the detailed molecular mechanism remains unclear.

The mechanism by which maoto attenuated the polyI:C-induced production of PG/LT seems to differ from that of non-steroidal anti-inflammatory drugs (NSAIDs). In normal rats, for instance, maoto did not have a consistent effect on metabolites of COXs and LOXs, whereas polyI:C upregulated metabolites of AA such as PGs and LTs, which are related to the induction of pyretic and inflammatory symptoms. COX products such as PGs did not change, but TXB2 increased after maoto administration. Concerning 5-LOX products, N-acetyl-LTE4 potently decreased, while 9S-hydroperoxy-10E,12Z-octadecadienoic acid (9-HpODE) increased. Recently, it was reported that individual NSAIDs have specific effects on broad lipid mediators. For example, the serum lipidomics of aspirin-treated and ibuprofen-treated patients showed a consistent decrease in various COX and 5-LOX products, including TXB2, whereas the effects on CYP products were relatively moderate.^33,34^ In addition, it was noted that the effects on 12-LOX and 15-LOX differed between aspirin and ibuprofen. Our study data suggested that maoto activated certain metabolic pathways, such as HETE and DHET biosynthesis, eventually leading to a decrease in AA supply in the COX-related and/or 5-LOX-related metabolic pathways (Fig. 6a, b, c﻿, Supplementary Fig. 8 and Supplementary Table 4). HpODE and DiOHM biosynthesis, which originates from linoleic acid (LA), was also enhanced, possibly reducing AA generation. In general, metabolites of ω-3 fatty acids are known as anti-inflammatory mediators. DHA inhibits proinflammatory cytokines, TNF-α and IL-6, and shows an anti-inflammatory effect via G protein-coupled receptor 120.^35^ In addition to the known anti-inflammatory properties of EPA and DHA, some of the lipid mediators induced by maoto such as 14,15-DHET and 20-HETE have been reported to exert anti-inflammatory effects via the activation of peroxisome proliferator-activated receptors.^36–40^ Thus, maoto seems to decrease PGs/LTs via interference in multiple loci upstream of the PG/LT biosynthesis pathway, and to exert its beneficial effect on inflammatory response by tilting the balance of AA metabolism toward DHET/HETE synthesis and away from PG/LT synthesis.

It is unclear by which compounds and by which mechanisms the profound and rapid (~2 h) effects of maoto on lipid mediator metabolism are induced. Among the ingredients analyzed in the present PK study, glycyrrhetinic acid is unlikely to be involved in these effects because it was produced and absorbed relatively slowly after maoto administration. Other maoto compounds such as ephedrines, cinnamic acid and amygdalin were rapidly absorbed, and therefore might be responsible for the effects on lipid mediator metabolism. Accordingly, methylephedrine is metabolized to ephedrine by CYP.^41^ Several compounds in GR such as isoliquiritigenin affect CYP.^42^ The effects of maoto compounds on various CYPs have scarcely been investigated, and the CYPs involved in AA metabolism have not been fully elucidated; however, it may be possible that the multifaceted effects of maoto constituents on various CYPs affect AA metabolism. Along this line, it should be noted that, although many AA-derived eicosanoid metabolites increased in polyI:C-treated rats, maoto administration led to a decrease in almost all eicosanoid metabolites at 4 h. This suggests that the three major metabolic pathways of eicosanoid biosynthesis were all downregulated by maoto. When we analyzed the effect of maoto on innate metabolites by non-targeted analysis, several lysophospholipids containing LA, α-LA, eicosadienoic acid and adrenic acid as fatty acid were increased 1 h after maoto treatment. Because lysophospholipids generally modulate the acute proinflammatory cytokine response,^43^ these phospholipids might be associated with modulation of the inflammatory response by maoto. Furthermore, in the of profiling of lipid mediators after maoto treatment, those downstream of the fatty acids detected in non-targeted analysis were increased (Fig. 6 and Supplementary Fig. 7). These results suggested that maoto affects the production of lysophospholipids upstream of lipid mediators, and affects a broad extent of lipid mediators. We have integrated these findings into a hypothesis of the multiple modes of action of maoto in Supplementary Fig. 1.

Furthermore, our analysis of common metabolites showed that metabolites of the TCA cycle were downregulated and those of PPP were upregulated in the plasma of polyI:C-treated rats. These observations might partially reflect the changes in metabolic status related to the accelerated proliferation of macrophages by activation of TLR ligands.^44^ In addition, polyI:C consistently decreased norepinephrine and increased adenosine. The increase in adenosine is in line with previous studies reporting an increase in circulating adenosine in TLR-mediated systemic inflammation.^45,46^ Adenosine is a signaling molecule that mediates numerous anti-inflammatory effects^47^ and is the final product of adenosine triphosphate breakdown; therefore, its presence might reflect both the onset of a negative feedback loop to limit inflammatory response^48^ and enhanced extracellular adenosine generation by ectoenzymes on the macrophage surface. Furthermore, administration of maoto to both polyI:C-treated and untreated rats increased ketone bodies, reflecting beta-oxidation. These changes, along with a concomitant rapid and transient increase in norepinephrine, are indicative of the sympathomimetic effect of EH in maoto. Moreover, maoto treatment led to a decrease in amino acids, including BCAAs, particularly in polyI:C-treated rats. These changes in BCAA and ketone bodies might signify altered energy metabolism because these products might act as alternative energy sources under limited glucose utilization.^49–51^ We also noted that maoto suppressed body weight loss without restoring food intake.

While we observed a relevant effect of maoto on acute inflammatory response in the study model, we also recognized the limitations of this study in explaining the entire effect of maoto. For instance, the antipyretic effect of maoto was not confirmed in the present polyI:C model, in contrast to the findings of a study using an influenza virus infectious model,^7^ suggesting that its antipyretic effect in an infectious model is not simply due to the inhibition of inflammatory cytokines. Furthermore, the beneficial effects of maoto on body weight loss could not be reproduced by any single herb treatment, although the improvement in locomotor activity was fully achieved by EH, which might be explained by the well-known sympathomimetic effect of ephedrines.^24,25^ This suggests that some of the effects of maoto require a combination of multiple herbs to act on multiple regulatory mechanisms in complex biological networks to produce an integrated effect at the level of the organism. To tackle these challenges, the combinatorial effect of components/compounds should be analyzed in several models, including influenza infection models.

Concerning the combination effect of maoto, it should be noted that the voluminous and diversified compounds in maoto, which are distributed in a “long-tail” fashion, might be involved in its unique pharmacological activities. A theoretical framework to explain how the systematic control of complex biological networks can be achieved via perturbations of long-tail components is being proposed.^52^ We conjecture that the contribution of each “tail” component (i.e., minor compound) might be small, but the sum of their diverse contributions might amount to levels of activity comparable to those of the head component (i.e., major “active” compound). For instance, well-known compounds in maoto, such as ephedrine, prunasin and glycyrrhetinic acid, are considered to comprise the “head” components. Investigating the potential contributions of “long-tail” components to the pharmacological effects of maoto relative to those of the “head” components might be critically important for elucidating the totality of the effects and mechanisms of maoto.

To address the combination effect of compounds, we would like to point out the importance of possible inter-herbal interactions at the stage of decoction and extraction.^53^ Because Kampo is not a combination of individually prepared herbal extracts, but rather an extract prepared from decocting a mixture of several raw herbs together, the overall extraction efficiency and compounds extracted from each herb are affected by the herbs that coexist in the decoction and extraction solution.

Another interesting possibility suggested by this study is that Kampo might exert its pharmacological activities by inducing metabolic perturbations in the host. The extraordinary number of compounds in Kampo might affect various sites and stages of the host metabolic pathways. Csete and Doyle^54^ have pointed out that the changes in host metabolism inevitably alter the context, circumstances and operating conditions of signaling and execution systems consisting of transcription factors and various modifications of signaling proteins. Thus, intervention at the level of host metabolism, which changes the “metabolic landscape” upon which the biological signaling systems exert their effect, might form part of the mechanism of action of herbal medicines.

Regarding the safety of maoto, cardiovascular events due to overdosing on ephedrine/ephedra herbs have been reported.^55^ Furthermore, while the detailed disposition and metabolism of natural compounds remains unclear, as described above maoto constituents possibly have profound effects on CYP450: for example, compounds in Glycyrrhizae radix are known to inhibit CYP450 isoforms.^42^ Therefore, while the direct inhibition of CYP450 isoforms by maoto administration has not been established, we need to carefully consider its metabolic interactions. Comprehensive profiling of herbal compounds is informative not only for efficacy but also for adverse events such as drug interactions.

In conclusion, we have shown that the extensive alterations in maoto compounds/metabolites before and after administration, and the profound and broad influence on host metabolism, are unequivocally relevant to its diverse pharmacological effects. Extensive profiling of compounds, metabolites and biological activities of THM and THM-treated hosts is thus a powerful and indispensable means for clarifying the beneficial effects and mechanisms of action of THM.

## Methods

The detailed protocols of each experiment are available under the “Supplementary Methods” section.

### Maoto and constitutive herbs

Maoto is an extracted mixture of Ephedrae Herba (EH) (32.3%), Armeniacae Semen (AS) (32.3%), Cinnamomi Cortex (CC) (25.8%), and Glycyrrhizae Radix (GR) (9.6%). The dry powdered extracts of maoto (supplied as TJ-27) and constituent herbs industrially produced by spray-drying were supplied by Tsumura & CO. (Tokyo, Japan).

### Profiling of compounds in maoto extract

Extracts of maoto and its constitutive herbs were analyzed by using LC-Orbitrap-MS. To identify the compounds, the obtained peaks were matched to reference peaks in public databases such as Human metabolome database (http://www.hmdb.ca) and KNApSAcK (http://kanaya.naist.jp/KNApSAcK/), as well as in-house databases of natural compounds at Kazusa DNA institute and TSUMURA Research Laboratories.

### Profiling of compounds in maoto and metabolites in rat plasma after administration of maoto

Maoto (2 g/kg dissolved in distilled water) was orally administered to male Sprague–Dawley (SD) rats, and whole blood was obtained from the abdominal inferior vena cava. Plasma samples were analyzed by non-targeted analysis using LC-Orbitrap-MS. The peaks of metabolites specific to the plasma of maoto-administered rats were identified by comparison to the spectra of control plasma. The chemical formulae and names of the detected peaks were estimated by collation with the databases described in the Supplementary Methods to identify their chemical compositions and structures.

### Analysis of PK properties of maoto-derived major compounds and metabolites

The PK properties of maoto-derived major compounds and metabolites after maoto administration (1, 2 and 4 g/kg) were determined by LC-MS/MS (Supplementary Table 2).

### Pharmacological profiling of maoto

The effects of maoto on polyI:C-induced acute inflammatory response and sickness phenotypes (i.e., changes in locomotor activity, body weight, food intake, and body temperature) were analyzed in male SD rats. Maoto (0.25, 0.5, 1.0, and 2.0 g/kg dissolved in distilled water) was orally administered concurrently with an intraperitoneal injection of polyI:C (3 and 6 mg/kg; dissolved in saline). Distilled water and saline were administered as vehicle to rats in each control group. The effects of maoto on polyI:C-induced sickness phenotypes, including decreased locomotor activity, body weight and food intake, as well as febrile response, were evaluated. Whole blood was obtained from the abdominal inferior vena cava. Plasma cytokine levels were measured by ELISA or multiplex immunoassay; common metabolites and lipid mediators were analyzed by GC-MS/MS and LC-MS/MS, respectively.

Pathway maps of lipid mediators were created by using CellDesigner 4.4 software (http://www.celldesigner.org). We evaluated the effects of the treatments on specific lipid mediators and categorized them according to COX, CYP and LOX pathways by Fisher’s exact test. The proinflammatory and anti-inflammatory indices were analyzed as described by Tam et al. with modification.^22^

### Data availability

Compound and metabolite data measured by LC-Orbitrap-MS were shown in Supplementary Fig. 2a–c. Metabolome data measured by GC-MS/MS and LC-MS/MS were shown in Supplementary Tables 3 and 4. Relevant experimental data are available from the authors.

## Electronic supplementary material

Supplementary Materials
