# Supplementary material for: Deconstructing the traditional Japanese medicine “Kampo”: compounds, metabolites and pharmacological profile of maoto, a remedy for flu-like symptoms
Source: NPJ Syst Biol Appl. 2017 Oct 24;3:32. doi: 10.1038/s41540-017-0032-1 (PMC5654968; doi:10.1038/s41540-017-0032-1)
Supplement: Supplementary file 1 — Supplementary Materials [file 41540_2017_32_MOESM1_ESM.pdf]

Supplementary Methods

Maoto extract

Maoto is an extracted mixture of Ephedrae Herba (EH), Armeniacae Semen (AS), Cinnamomi Cortex (CC), and Glycyrrhizae Radix (GR), combined in a ratio of 10 (32.3%) : 10 (32.3%) : 8 (25.8%) : 3 (9.6%). The dry powdered extracts of maoto (Lot No.331010700/ 341115500), its constituent herbs (Lot No. EH, 2091037010; CC, 2121003010; AS, 2141046010; GR, 2131013010), and maoto analog, a mixture of three constituent herbs of maoto (CC, AS and GR) without EH (termed EH(-)-maoto (2160027030)) produced by spray-drying, were supplied by Tsumura & CO. (Tokyo, Japan).

Animals

Male Sprague–Dawley (SD) rats were purchased from Japan SLC, Inc. (Shizuoka, Japan) at 7 weeks of age, and used from 8 weeks of age after habituation. Rats were housed individually in a cage with paper chips, and permitted free access to food and water. The rearing conditions were kept at a room temperature of 23°C, relative humidity of 60%, and 12 h light-dark cycle

(7:00-19:00). Rats were maintained and used for the experiments in accordance with the Guidelines for the Care and Use of Laboratory Animals of Tsumura & CO. All experimental procedures were carried out upon approval from the Laboratory Animal Committee of Tsumura & CO.

Non-targeted and targeted analysis of maoto compounds

Blood sampling

Maoto (1, 2, and 4 g/10 mL/kg) (dissolved in distilled water) was orally administered to 16-h fasted rats (n=3). Rats were anesthetized with isoflurane (AbbVie GK, Tokyo, Japan) before blood sampling, and whole blood was withdrawn through the abdominal inferior vena cava with a heparinized syringe at 0.25, 0.5, 1, 2, 4, 6, 8, 10, and 24 h after maoto/vehicle administration. Plasma was obtained by centrifugation at 1,700 x g for 15 min at 4°C and stored at -80°C until use.

Non-targeted analysis by LC-Orbitrap Mass Spectometry (MS)

One hundred microliters of plasma sample or 1 mg of maoto extract powder were extracted with 300 μL of methanol or 1 mL of 75% MeOH (aq). For

non-targeted metabolome analysis, LC–MS was performed using an Agilent 1200 HPLC system (Agilent Technologies, Santa Clara, CA) coupled to an LTQ Orbitrap XL–MS system (Thermo Fisher Scientific Inc., San Jose, CA), equipped with an electrospray source operating in positive-ion mode. The spray voltage and capillary temperature were 4 kV and 300°C, respectively. The analysis consisted of two scan events. Scan Event 1 was a full mass type (Analyzer, FTMS; Resolution, 60,000). Scan Event 2 was a MS/MS type (Analyzer, Ion Trap MS; Act Type, collision-induced dissociation; Normalized Collision Energy, 35.0%). An aliquot of the extracted sample (5 μl) was injected into a TSKgel ODS-100V reversed-phase column (column size, 3.0 × 50 mm; particle size, 5.0 μm; TOSOH Corporation, Tokyo, Japan). The column temperature was set at 40°C. Mobile phases A (0.1% formic acid) and B (acetonitrile including 0.1% formic acid) were used with a gradient of 3% to 97% B from 0 to 15 min, 97% B from 15 to 20 min, 97% to 3% B from 20 to 20.1 min, and 3% B for 4.9 min before the next injection, at a flow rate of 0.4 μL/min.

The data were acquired with X-Calibur software (Thermo Fisher Scientific Inc.,

San Jose, CA) and were exported in text files using MSGet software (http://www.kazusa.or.jp/komics/software/MSGet; Kazusa DNA Research Institute, Chiba, Japan). LC–MS data were analyzed with PowerGet software (http://www.kazusa.or.jp/komics/software/PowerGet/ja/; Kazusa DNA Research Institute) by using previously described methods.[1] Briefly, PowerGet is a Java software package for detection, alignment, and annotation of metabolite features from data obtained using LC/high-resolution MS. These data were used to calculate changes relative to the control group. Differences between groups were compared using the Welch's t-test.

To identify compounds and metabolites that were specifically detected in the plasma of maoto-administered rat, we extracted peaks that were not present in control plasma from naïve rats. Chemical formulae were estimated from the accurate mass of the detected peak measured by mass analysis, coupled with database collation (KEGG (http://www.kegg.jp), KNApSAcK (http://kanaya.naist.jp/KNApSAcK_Family/), LipidMAPS (http://www.lipidmaps.org), Flavonoid Viewer

(http://webs2.kazusa.or.jp/mfsearcher/flavonoidviewer/), and Human metabolome database (http://www.hmdb.ca) using MF Searcher software (Kazusa DNA Research Institute).[1] A list of identified chemical formulae was prepared, and the chemical names were predicted from MS/MS information and the Tsumura crude drug database.

To clarify the distribution of the compounds detected in rat plasma after oral maoto administration (2 g/kg), the peaks of chemical composition-determined (CCD) compounds in non-targeted analysis were arranged in descending order of peak intensity. The compounds detected in plasma by targeted analysis were also arranged in descending order of concentration.

Analysis of the PK properties of major maoto compounds by LC-MS/MS

Pharmacokinetic properties of compounds in maoto after its administration (1, 2 and 4 g/10mL/kg) were determined by LC-MS/MS. Based on a literature search, 21 compounds were selected for analysis as major constituents of maoto: amygdalin, prunasin, scopoletin, *l*-ephedrine, *d*-pseudoephedrine,

dl-methylephedrine, (+)-catechin, epicatechin, epigallocatechin, (*E*)-cinnamic acid, procyanidin B2, procyanidin C1, 7-hydroxycoumarin, 18β-glycyrrhetinic acid, liquiritin, liquiritigenin, liquiritin apioside, isoliquiritin, isoliquiritigenin, isoliquiritin apioside, and glycycoumarin. 18β-Glycyrrhetinic acid was purchased from Sigma-Aidrich (St. Louis, MO, USA); the other compounds were supplied by Tsumura & CO.

For quantification of *l*-ephedrine, *d*-pseudoephedrine, and *dl*-methylephedrine, 25 µL of standard solution and 25 µL of internal standard solution were mixed with 200 µL of diluted plasma sample. Ethyl acetate (250 µL) was then added to the solution, followed by mixing and centrifugation (7,000 g, 5 min). The supernatant was collected and dried at 40°C under a stream of nitrogen gas. The dried residue was dissolved in 50 µL of the HPLC mobile phase used for each analytical method, and a 10-µL aliquot was injected into the LC-MS/MS systems. The quantification of amygdalin, prunasin, (*E*)-cinnamic acid, glycyrrhetic acid, liquiritin, liquiritin apioside, liquiritigenin, isoliquiritin, isoliquiritin apioside, and isoliquiritigenin was carried out by the same procedure

except that methanol (750 μL) was used as the extraction solution instead of ethyl acetate.

An Agilent 1100 HPLC system (Agilent Technologies) coupled to an API4000 triple quadrupole mass spectrometer fitted with a Turbo IonSpray electrospray ionization instrument (AB Sciex, Tokyo, Japan) was used for mass spectrometry and detection of 18β-glycyrrhetinic acid, liquiritin, liquiritin apioside, liquiritigenin, isoliquiritin, isoliquiritin apioside, isoliquiritigenin, and glycycoumarin. Analyst Software, Ver.1.6.2 (AB Sciex) was used for data acquisition. Ten microliters of extracted sample was injected into the HPLC system. An Inertsil Ph-3 column (column size, 2.1 × 100 mm; particle size, 3.0 μm; GL sciences, Tokyo, Japan) was used to separate each analyte at 40°C. The mobile phase consisted of solution A (10 mM ammonium acetate) and solution B (acetonitrile) with a gradient of solution B (20%, 0 min; 20%, 1 min; 65%, 13 min; 20%, 13.01 min; and 20 %, 18 min) at a flow rate of 0.3 mL/min. The mass spectrometer was operated in negative ion mode. The high-purity nitrogen gas comprised ion source gas 1 (GAS1), ion source gas 2 (GAS2),

curtain gas (CUR) and collision-activated dissociation gas (CAD) at pressures of 50, 50, 20, and 9 psi, respectively. The optimized Turbo IonSpray voltage (IS) and temperature (TEM) were set at -4500 V and 600°C, respectively. Each analyte was quantified by the multiple reaction monitoring (MRM) transition of *m*/*z* (Q1, Q3; 18β-glycyrrhetinic acid, 469.2, 425.3; liquiritin, 417.0, 254.8; liquiritin apioside, 549.2, 135.0; liquiritigenin, 254.9, 118.9; isoliquiritin, 417.0, 255.0; isoliquiritin apioside, 549.2, 254.9; isoliquiritigenin, 254.9, 119.0; and glycycoumarin, 367.0, 308.7) in a single analytical run.

An Agilent 1290 HPLC system (Agilent Technologies) coupled to a TripleQuad6500 triple quadrupole mass spectrometer fitted with a Turbo IonSpray electrospray ionization instrument (AB Sciex) was used for mass spectrometry and detection of (+)-catechin, epicatechin, epigallocatechin, procyanidin B2, procyanidin C1, and 7-hydroxycoumarin. Analyst Software, Ver.1.6.2 (AB Sciex) was used for data acquisition. Ten microliters of extracted sample was injected into the HPLC system. An Ascentis ExpressRP-amide column (column size, 2.1 × 100 mm; particle size, 2.7 μm; Supelco analytical

Inc., Tokyo, Japan) was used to separate each analyte at 40°C. The mobile phase consisted of solution A (0.2% ammonium acetate) and solution B (acetonitrile containing 0.2% ammonium acetate) with a gradient of solution B (12%, 0 min; 12%, 5 min; 88%, 13 min; 12%, 13.01 min; and 12%, 18 min) at a flow rate of 0.25 mL/min. The mass spectrometer was operated in positive ion mode. The high-purity nitrogen gas comprised GAS1, GAS2, CUR, and CAD at pressures of 50, 20, 30, and 8 psi, respectively. The optimized Turbo IS and TEM were set at 5500 V and 500°C, respectively. Each analyte was quantified by the MRM transition of *m*/*z* (Q1, Q2; (+)-catechin, 291.0, 139.0; epicatechin, 291.0, 139.0; epigallocatechin, 307.1, 139.1; procyanidin B2, 579.1, 291.0; procyanidin C1, 867.2, 579.0; and 7-hydroxycoumarin, 163.1, 77.0) in a single analytical run.

An Agilent 1290 HPLC system (Agilent Technologies) coupled to a TripleQuad6500 triple quadrupole mass spectrometer fitted with a Turbo IonSpray electrospray ionization instrument (AB Sciex) was used for mass spectrometry and detection of amygdalin, prunasin, scopoletin, and €-cinnamic

acid. Analyst Software, Ver.1.6.2 (AB Sciex) was used for data acquisition. Ten microliters of extracted sample was injected into the HPLC system. An Ascentis ExpressRP-amide column (column size, 2.1 × 100 mm; particle size, 2.7 μm; Supelco analytical Inc., Tokyo, Japan) was used to separate each analyte at 40°C. The mobile phase consisted of solution A (0.2% ammonium acetate) and solution B (acetonitrile containing 0.2% ammonium acetate) with a gradient of solution B (12%, 0 min; 12%, 10 min; 95%, 10.1 min; 95%, 15 min; 12%, 15.1 min; and 12%, 20 min) at a flow rate of 0.25 mL/min. The mass spectrometer was operated in positive and negative ion mode. The high-purity nitrogen gas comprised GAS1, GAS2, CUR, and CAD at pressures of 50–60, 60–80, 10–30, and 7–10 psi, respectively. The optimized Turbo IS and TEM were set at -4000–5500 V and 500–700°C, respectively. Each analyte was quantified by the MRM transition of *m*/*z* (Q1, Q3; amygdalin, 456.1, 323.1; prunasin, 294.0, 161.0; scopoletin, 193.1, 133.0; and (*E*)-cinnamic acid, 146.9, 103.0) in a single analytical run.

An Agilent 1290 HPLC system (Agilent Technologies) coupled to a

TripleQuad6500 triple quadrupole mass spectrometer fitted with a Turbo IonSpray electrospray ionization instrument (AB Sciex) was used for mass spectrometry and detection of *l*-ephedrine, *d*-pseudoephedrine, and *dl*-methylephedrine. Analyst Software, Ver.1.6.2 (AB Sciex) was used for the data acquisition. Ten microliters of extracted sample was injected into the HPLC system. An Inertsil Ph-3 column (column size, 2.1 × 100 mm; particle size, 3.0 μm; GL Sciences) was used to separate each analyte at 40°C. The mobile phase consisted of solution A (0.2% formic acid) and solution B (acetonitrile) with a gradient of solution B (2%, 0 min; 2%, 9 min; 90%, 13 min; 2%, 13.01 min; and 2%, 18 min) at a flow rate of 0.3 mL/min. The mass spectrometer was operated in positive ion mode. The high-purity nitrogen gas comprised GAS1, GAS2, CUR, and CAD at pressures of 50, 40, 30, and 8 psi, respectively. The optimized Turbo IS and TEM were set at 4500 V and 600°C, respectively. Each analyte was quantified by the MRM transition of *m*/*z* (Q1, Q3; *l*-ephedrine, 166.2, 133.1; *d*-pseudoephedrine, 1662, 133.1; and *dl*-methylephedrine, 180.1, 162.1) in a single analytical run.

Plasma PK data were analyzed by noncompartmental modeling using Phoenix WinNonlin (version 6.3, Certara L.P., St. Louis, MO, USA) to determine various PK constants including C_{max}, t_{max}, $t_{1/2}$, and $AUC_{0–last}$. The $t_{1/2}$ was divided by $log_e 2/k_e$, where k_e is the rate constant of terminal elimination (at least three data points on the descending linear limb).

Pharmacological study of maoto

Administration of maoto, constituent herbs and polyI:C

PolyI:C sodium salt (Sigma-Aldrich) (6 mg/10 mL/kg) was dissolved in saline (Otsuka Pharmaceutical Co, Tokyo, Japan) and administered by intraperitoneal injection. Maoto (0.25, 0.5, 1 and 2 g/10 mL/kg) and its constituent herbs EH (645 mg/kg), AS (645 mg/kg), CC (516 mg/kg) and GR (194 mg/kg) (amounts equivalent to those in 2 g/kg of maoto) were dissolved in distilled water, and orally administered concurrently with intraperitoneal injection of polyI:C (dissolved in saline) to rats. Distilled water and saline, respectively, were administered as vehicle to rats in each control group.

To evaluate the effects of EH(-)-maoto, polyI:C supplied by InvivoGene, (PolyI:C(HMW), San Diego, CA, USA) (3 mg/10 mL/kg) was dissolved in saline and administered by intraperitoneal injection with oral administration of EH(-)-maoto (1355 mg/kg) as described above.

Measurement of locomotor activity, body weight, and rectal temperature

Nocturnal spontaneous activity of rats was measured in home-cages by using an infrared sensor NS-AS01 (Neuroscience, Tokyo, Japan) as an index of activity during the active phase of rats. After drug administration during the light phase, the sensor counts were measured during the nocturnal phase from 19:00 on the treatment day to 07:00 on the following day. The activity score after treatment was reported as the percentage change from the score immediately before the treatment. Changes in body weight and food intake were assessed one day after treatment. The temperature of the rats was measured from 1 h to 6 h after treatment by inserting a temperature sensing probe (BWT-100A/HS-2, Bio Research Center, Nagoya, Japan) into the rectum to a depth of 2.0 cm. To validate our experimental paradigm, we first measured sickness phenotypes in a

polyI:C model and confirmed that significant changes were induced by polyI:C as compared with a control; we then used this experimental model to evaluate the effects of maoto on sickness phenotypes. If any outliers in the data set were determined to be due to artifacts, they were excluded from the analysis. Data are presented as mean ± SEM. Statistical significance of the data was determined by Welch's t-test with Bonferroni correction or ANOVA with Bonferroni's multiple comparisons test. The significance level in each statistical analysis was set at $P<0.05$.

Blood sampling

Treated rats were anesthetized with isoflurane (AbbVie GK), and blood was collected from the inferior vena cava with EDTA・2K (Wako Pure Chemical Industries, Osaka, Japan). Blood samples were obtained at 1, 2, 4, 6 and 20 h after treatment, and centrifuged at 1,200 x g at 4°C for 30 min to prepare plasma samples, which were then stored at -80°C until use.

Cytokine measurement by enzyme-linked immunosorbent assay (ELISA) and multiplex immunoassay

The concentrations of TNF-α and IL-6 in plasma samples were evaluated by ELISA (R&D Systems, Minneapolis, MN, USA). Multiplex immunoassays (Milliplex, Merck KGaA, Darmstadt, Germany) were performed to quantify the concentrations of IL-1β, IL-10 and INF-γ. Assays were performed according to the manufacturer's protocol. To validate our experimental paradigm, we first measured cytokine concentrations in a polyI:C model and confirmed that significant changes in concentrations were induced by polyI:C as compared with the control; we then used this experimental model to evaluate the effects of maoto on cytokine expression. If any outliers in the data set were determined to be due to artifacts, they were excluded from the analysis. Data are presented as mean ± SEM. Statistical significance of the data was determined by Welch's t-test with Bonferroni correction. The significance level in each statistical analysis was set at $P<0.05$.

Analysis of metabolites by GC-MS/MS

Plasma samples obtained at 1, 2, 6 and 20 h after treatment were used for analysis. To extract low molecular weight metabolites for GC-MS/MS analysis,

50 μL of plasma was mixed with 260 μL of a solvent mixture (MeOH:H2O:CHCl3 = 2.5:1:1) containing 10 μL of 0.5 mg/mL 2-isopropylmalic acid (Sigma-Aldrich) dissolved in distilled water; the solution was shaken at 1,400 rpm for 30 minutes at 37°C before centrifugation at 19,000 × g for 3 minutes at 4°C. Next, 150 μL of the resulting supernatant was transferred to a clean tube and 140 μL of distilled water was added. After mixing, the solution was centrifuged at 19,000 × g for 3 minutes at 4°C, and 180 μL of the supernatant was transferred to a clean tube and lyophilized by using a freeze dryer. For oximation, 80 μL of 20 mg/mL methoxyamine hydrochloride (Sigma-Aldrich) dissolved in pyridine was mixed with the lyophilized sample before sonication for 20 min in a water bath sonicator. The samples were then shaken at 1,200 rpm for 90 minutes at 30°C. Next, 40 μL of N-methyl-N-trimethylsilyl-trifluoroacetamide (MSTFA) (GL Science) was added for derivatization, and the mixture was incubated at 1,200 rpm for 30 minutes at 37°C. The mixture was then centrifuged at 19,000 × g for 3 minutes and the resulting supernatant was applied to GC-MS/MS.

GC-MS/MS was performed using a GCMS-TQ8040 instrument

(Shimadzu, Kyoto, Japan) with a fused silica capillary column (DB-5; inner diameter, 30 m × 0.25 μm; film thickness, 1 μm; Agilent Technologies). Chromatogram acquisition, detection of mass spectral peaks, and their waveform processing were performed using Shimadzu GCMSsolution software. Identification of low molecular weight metabolites was performed by using the Smart Metabolites Database (Shimadzu), which contains a mass spectral library, method files specifying the above-described analytical conditions, and data analysis parameters for 475 compounds such as amino acids, fatty acids, and organic acids. The peak intensity of each quantified ion was calculated and normalized to that of 2-isopropylmalic acid, which was used as an internal standard.

Analysis of lipid mediators by LC-MS/MS

Lipid mediators were measured in plasma sampled at 2 h and 4 h after treatment. To extract low molecular weight metabolites for LC/MS analysis, 1 ml of methanol with an internal standard mixture (standards obtained from Cayman Chemical, Ann Arbor, MI, USA) was mixed with 100 μl of plasma sample for 5

min at room temperature and then centrifuged at 15,000 x g for 3 min. The supernatant was diluted with 4 ml of 0.1% formic acid in water and gently mixed. The mixture was loaded onto a preconditioned solid-phase extraction cartridge (STRATA-X, 10 mg/1 ml, Phenomenex, Torrance, CA, USA), which was then washed with 1 ml each of 0.1% formic acid and 15% ethanol. The lipids were eluted with 250 μl of 0.1% formic acid in methanol, and the eluent was evaporated by vacuum evaporator and reconstituted in 20 μl of methanol. Five microliters of sample was injected for analysis. The LC/MS system consisted of two LC-30AD pumps, an SIL-30AC auto-sampler, a CTO-20A column oven, a CBM-20A system controller, and a triple quadrupole mass spectrometer LCMS-8050 (Shimadzu). A reversed-phase column (Kinetex C8, 2.1 x 150 mm, 2.6 μm, Phenomenex) was used for chromatographic separation. Chromatogram acquisition, detection of mass spectral peaks, and their waveform processing were performed by using LCMSsolution software and LC-MS/MS Method Package for Lipid Mediators Ver.2 (Shimadzu), which contains a mass spectral library, method files specifying the analytical conditions,

and data analysis parameters for 158 lipid mediators derived from arachidonic acid, eicosapentaenoic acid or docosahexaenoic acid, and so on. The peak area of each quantified ion was calculated and normalized to those of the internal standard mixture containing 0.5 ng/μL each of tetranor-PGEM-d6, TXB2-d4, PGE2-d4, PGD2-d4, LTC4-d5, LTB4-d4, 5-HETE-d8 and 15-HETE-d8, 0.25 ng/μL of oleoylethanolamide (OEA)-d4, and 10 ng/μL of AA-d8 in methanol.

Processing, analysis and visualization of metabolomics data

Processing of metabolomics data was performed with MetaboAnalyst 3.0 software (http://www.metaboanalyst.ca). Missing values in the raw data were replaced by half of the minimum positive value, and these data were used for subsequent statistical analysis. Pathway maps of lipid mediators were created with CellDesigner 4.4 software (http://www.celldesigner.org) and referenced public pathway databases, including KEGG, PANTHER (http://pantherdb.org/about.jsp), LIPID MAPS, and the Human Metabolome database, as well as publications such as Harkewicz et al, Buczynski et al., Dennis et al., Tam et al., and Yamada et al. [2,3,4,5,6] Metabolomics data were

mapped to visualize the relationship between each metabolite. If outliers in the data set were determined to be due to artifacts, they were excluded from the analysis.

Effect of polyI:C/maoto treatment on metabolic pathway of lipid mediators

We evaluated whether lipid mediators categorized by the COX, CYP and LOX pathways were specifically affected by maoto treatment by using Fisher's exact test. Metabolites that significantly decreased or increased relative to the control group ($P<0.05$ by Welch’s t-test), and all mediators metabolized in the COX, CYP and LOX pathways that were detected 2 h after treatment were included in the analysis. Metabolites detected in each metabolic pathway that significantly increased or decreased are shown in Supplementary Information of Figure 6d.

Effect of polyI:C/maoto on proinflammatory and anti-inflammatory indices

The proinflammatory and anti-inflammatory indices were calculated and categorized as described by Tam et al. [7] with modification, as shown in Supplementary information of Figure 6e and 6f.

The fold change in each lipid mediator was calculated relative to the median of control rats. Mediators with values of 2 or higher were included in the analysis. The fold changes in each mediator were normalized to the maximum value across all samples. The maximum value of each lipid mediator was normalized to one. We added the value of each sample within the proinflammatory and anti-inflammatory groups to obtain the total activity. To generate the index at each time point, the percentage of proinflammatory and anti-inflammatory mediator activity was calculated by dividing the measured activity by the maximum possible activity in each group. The score of each group was normalized to the score of its own control group. Data are presented as mean ± SEM. Statistical significance of the data was determined by Welch's t-test with Bonferroni correction. The significance level in each statistical analysis was set at $P<0.05$.

Supplementary References

1. Sakurai, N. *et al.* Tools and databases of the KOMICS web portal for preprocessing, mining, and dissemination of metabolomics data. *Biomed. Res. Int.* **2014**, 194812 (2014).
2. Harkewicz, R., Fahy, E., Andreyev, A. & Dennis, E. A. Arachidonate-derived dihomoprostaglandin production observed in endotoxin-stimulated macrophage-like cells. *J. Biol. Chem*. **282**, 2899-2910 (2007).
3. Buczynski, M. W., Dumlao, D. S. & Dennis, E. A. Thematic Review Series: Proteomics. An integrated omics analysis of eicosanoid biology. *J. Lipid. Res*. **50**, 1015-1038 (2009).
4. Dennis, E. A. *et al.* A mouse macrophage lipidome. *J. Biol. Chem*. **285**: 39976-39985 (2010).
5. Tam, V. C. Lipidomic profiling of bioactive lipids by mass spectrometry during microbial infections. *Semin. Immunol.* **25**, 240-248 (2013).
6. Yamada, M. *et al.* A comprehensive quantification method for eicosanoids and related compounds by using liquid chromatography/mass spectrometry

with high speed continuous ionization polarity switching. *J. Chromatogr. B Analyt. Technol. Biomed. Life. Sci.* **995-996**, 74-84 (2015).

7. Tam, V. C., Quehenberger, O., Oshansky, C. M., Suen, R., Armando, A. M. & Treuting, P. M. Lipidomic profiling of influenza infection identifies mediators that induce and resolve inflammation. *Cell* **154**, 213-227 (2013).
8. Drasar, B. S. & Hill, M. J., Human Intestinal Flora, 63, p. 61, (Academic press, London, 1974).

Supplementary Figure 1

Overview of the maoto study.

Overview of the comprehensive analysis and profiling of compounds and metabolites in rat plasma after maoto administration, and pharmacological profiling of the effect of maoto on flu-like symptoms are summarized. An integrated hypothesis of the mode of action of maoto is also shown.

Supplementary Figure 2

Profiles of metabolites detected in rat plasma at 1 h and 8 h after administration of maoto (2 g/kg). Categorization of the chemical formulae of (a) maoto-original and maoto-derived metabolites, (b) endogenous metabolites, and (c) undetermined metabolites, corresponding to the pie chart in Figure 2a is shown.

Supplementary Figure 3

Pharmacokinetic properties of major maoto compounds in rat plasma

after administration of maoto (2 g/kg).

The PK properties of 19 major maoto compounds are shown; 7-hydroxycoumarin and procyanidin C1 were not detected after maoto administration (2 g/kg). Compounds derived from Ephedrae Herba (EH), Armeniacae Semen (AS), Cinnamomi Cortex (CC) and Glycyrrhizae Radix (GR) are marked in red, blue, green and grey, respectively. Data represent mean ± SD (n=3 for each time point).

Supplementary Figure 4

Metabolic reactions of maoto-derived metabolites.

The major metabolic reactions of maoto-derived compounds detected by non-targeted and targeted analysis are summarized. Glycosides including glycyrrhizin, liquiritin, isoliquiritin and amygdalin are metabolized to their aglycon forms as gycyrrhetinic acid, liquiritigenin, isoliquiritigenin, and mandelonitrile, respectively. Glycyrrhizin is metabolized by α-glucuronidase, while liquiritin, isoliquiritin and amygdalin are metabolized by β-glucosidase.[8] The compounds

detected in maoto extract, rat plasma at 1 h, and rat plasma at 8 h after maoto administration (2 g/kg) are shown as salmon pink, light blue and mint green, respectively.

Supplementary Figure 5

Effects of maoto in naïve rats.

(a) Percentage change in the locomotor activity of each group compared with activity immediately before administration, and (b) changes in body weight, and (c) food intake 1 day after treatment. Maoto was administered at a dose of 2 g/kg, and saline was intraperitoneally administered. Data represent mean ± SEM. Control (n=10), Maoto (n=9). * $P<0.05$ versus control group by Welch's t-test.

Supplementary Figure 6

Effects of a mixture of three constituent herbs of maoto other than Ephedrae Herba (EH) on the polyI:C (PIC)-induced decrease in locomotor activity and body weight.

Effects of a mixture of three constituent herbs of maoto other than EH on polyI:C (PIC)-induced decrease in locomotor activity and body weight.

(a) Percentage change in locomotor activity, (b) change in body weight, (c) normalized data of changes in locomotor activity induced by PIC treatment in the control group (experiment 1 and experiment 2 are data from figure 3a and supplementary figure 6a, respectively), and (d) normalized data of body weight changes induced by PIC treatment in the control group (experiment 1, experiment 2, experiment 3 and experiment 4 are data from figure 3a, supplementary figure 6a, figure 3b and supplementary figure 6b, respectively). The score of each group is shown relative to the score immediately before administration. PIC was administered at a dose of 3 mg/kg. Maoto (MT) was administered at a dose of 2 g/kg, and maoto analog (EH(-)-maoto) was administered at a dose equivalent to the respective content of the three herbs in 2 g/kg of maoto (1355 mg/kg). Data represent mean ± SEM. For a and b, the following measurements were made: Control (n=5), PIC (n=6 for locomotor activity, and n=7 for body weight), PIC+MT (n=7), PIC+(EH(-)-maoto) (n=5); **

P<0.01, * P<0.05 versus control group; $^{++}$ P<0.01, $^{+}$ P<0.05 versus PIC group; $^{\#\#}$ P<0.01 versus PIC+Maoto group by ANOVA with Bonferroni's multiple comparisons test.

Supplementary Figure 7

Effect of maoto on polyI:C-induced cytokine response.

(a) TNF-α and (b) IL-10. PolyI:C and maoto were administered at doses of 6 mg/kg and 0.25, 0.5, and 1 g/kg, respectively. The concentrations of individual cytokines in rat plasma obtained from blood samples at 2 h after treatment were analyzed. Data represent mean ± SEM (n=10). ** P<0.01, * P<0.05 versus control group; $^{++}$ P< 0.01, $^{+}$ P<0.05 versus polyI:C group by Welch's t-test with Bonferroni correction.

Supplementary Figure 8

Heat map of changes in lipid mediator levels in polyI:C- and/or maoto-treated rats.

Heat map showing the fold change of fatty acids and lipid mediators in plasma from polyI:C-treated control (P/C), maoto-treated control (M/C), and maoto-treated polyI:C-treated (PM/P) rats. Fatty acids and lipid mediators categorized in accord with the pathway maps in Figure 5 are colored in gray. Magenta indicates an increase in metabolite fold number or upregulation; green indicates downregulation of a specific metabolite versus the control. The following measurements were made: at 2 h, Control (n=18), Maoto (n=17), PIC (n=17), PIC+Maoto (n=17); at 4 h, n=10.

Supplementary Table 1 Plasma pharmacokinetic parameters of compounds in maoto after oral administration of maoto at doses of 1, 2, and 4 g/kg

Compounds	Herb	1 g/kg				2 g/kg				4 g/kg			
		C_{max} (ng/mL)	T_{max} (h)	$t_{1/2}$ (h)	AUC_{last} (ng h/mL)	C_{max} (ng/mL)	T_{max} (h)	$t_{1/2}$ (h)	AUC_{last} (ng h/mL)	C_{max} (ng/mL)	T_{max} (h)	$t_{1/2}$ (h)	AUC_{last} (ng h/mL)
l-Ephedrine	EH	311	0.250	1.35	730	377	1.00	1.44	1560	545	0.250	4.19	2850
d-Pseudoephedrine		136	0.250	5.82	183	163	0.250	6.58	559	253	0.250	3.86	1180
dl-Methylephedrine		16.6	0.250	5.96	18.7	22.5	0.250	1.99	46.0	37.0	0.250	11.5	94.9
(+)-Catechin		BQL	-	-	BQL	0.443	1.00	-	0.111	3.75	0.250	-	1.71
Epicatechin		1.82	0.500	5.05	4.24	1.95	0.250	2.03	2.78	8.34	0.250	3.49	6.92
Epigallocatechin		0.197	0.500	5.23	0.457	0.103	0.500	3.16	0.260	0.456	0.250	0.834	0.286
Cinnamic acid	CC	1060	0.250	-	624	1280	0.250	1.30	2060	2120	0.250	1.64	5750
Procyanidin B2		BQL	-	-	BQL	0.687	1.00	-	0.172	4.64	0.250	-	2.06
Procyanidin C1		BQL	-	-	BQL	BQL	-	-	BQL	3.90	0.250	-	1.69
7-hydroxycoumarin		BQL	-	-	BQL	BQL	-	-	BQL	BQL	-	-	BQL
Amygdalin	AS	101	0.250	1.31	346	653	1.00	0.937	1520	5250	0.250	2.47	7610
Prunasin		2020	0.500	0.891	5520	2240	0.500	2.45	11900	3250	2.00	3.21	32000
Scopoletin		0.216	0.250	-	0.0700	0.425	0.250	2.39	0.428	0.237	0.250	5.44	0.591
Glycyrrhetinic acid	GR	234	8.00	-	2240	373	10.0	-	4510	301	8.00	-	3950
Glycycoumarin		0.0836	0.250	-	0.0105	0.0906	1.00	-	0.0227	0.0753	0.250	-	0.0580
Liquiritin		3.04	0.250	1.95	8.90	7.23	1.00	2.23	22.4	71.7	0.250	4.34	61.4
Liquiritigenin		18.0	0.250	11.9	17.4	15.1	1.00	-	35.0	16.1	0.250	3.06	84.4
Liquiritin apioside		3.07	2.00	3.96	12.9	7.24	1.00	2.62	31.7	169	0.250	3.85	130
Isoliquiritin		1.60	0.250	2.30	2.09	3.60	1.00	-	6.03	8.71	0.250	3.14	12.6
Isoliquiritigenin		10.4	0.250	3.42	19.8	10.1	0.250	11.1	31.0	7.59	0.250	3.61	58.9
Isoliquiritin apioside		0.355	0.250	-	0.113	1.49	1.00	-	3.16	9.08	0.250	0.891	8.10

BQL; below quantifiable limit
-: not determined

Supplementary Table 2 Number of compounds detected by non-targeted analysis in maoto extract and rat plasma after oral administration of maoto (2 g/kg)

	Maoto extract	Plasma after oral administration*	
		1h	8h
Compound peak	552	208 (18)	56 (10)
CCD compounds	352	89 (13)	36 (9)
CSD compounds	88	22 (7)	9 (3)

*Number of compound specifically detected in the plasma a:er maoto administration against the blank plasma are shown in the table.
The number in parentheses indicate the number of original compounds derived from maoto extract.
CCD compounds : Chemical composition determined compounds
CSD compounds : Chemical structure determined compounds

Supplementary Table 3 Fold changes and p values for common metabolites in poly I:C and/or maoto-treated rats

	P/C								M/C								PM/P							
	1h		2h		6h		20h		1h		2h		6h		20h		1h		2h		6h		20h	
**Compound name**	log_2FC	*p* value	log_2FC	*p* value	log_2FC	*p* value	log_2FC	*p* value	log_2FC	*p* value	log_2FC	*p* value	log_2FC	*p* value	log_2FC	*p* value	log_2FC	*p* value	log_2FC	*p* value	log_2FC	*p* value	log_2FC	*p* value
3-Phosphoglyceric acid	0.39	0.8372	1.16	0.4662	-0.24	0.6605	-0.66	0.1719	-4.04	0.3371	0.29	0.7390	-0.82	0.0684	0.12	0.8738	-1.67	0.4952	-0.33	0.8162	-0.59	0.4674	0.2803	0.5787
Pyruvic acid	0.03	0.9545	0.22	0.3542	0.26	0.6187	0.12	0.8512	0.52	0.3666	0.57	0.0616	-0.47	0.3832	0.87	0.1380	0.12	0.8229	0.58	0.1087	0.14	0.7386	-0.5492	0.3819
Fructose	0.03	0.8958	0.21	0.1273	-0.18	0.1543	0.15	0.1593	0.39	0.0549	***0.32***	***0.0017***	-0.09	0.4802	0.12	0.1438	0.04	0.8785	***-0.33***	***0.0351***	0.01	0.9237	-0.0785	0.3942
Glucose 6-phosphate	0.23	0.2384	0.01	0.9671	0.21	0.5344	-0.47	0.0634	0.26	0.4537	-0.21	0.3791	0.09	0.6588	0.26	0.4553	***-0.57***	***0.0247***	0.20	0.7317	-0.45	0.2118	0.1836	0.4307
Glucose	-0.02	0.8549	-1.38	0.3409	-0.09	0.1602	0.02	0.7020	0.08	0.4740	-1.04	0.4201	0.03	0.7072	0.02	0.5764	0.04	0.6017	0.17	0.2512	0.08	0.3071	0.0570	0.3503
Isocitric acid	-0.16	0.3763	0.41	0.2066	-0.03	0.8071	-0.22	0.0541	0.43	0.2338	***0.29***	***0.0378***	-0.13	0.4184	0.05	0.7109	0.22	0.2962	-0.04	0.8961	-0.05	0.7049	0.1426	0.1789
Oxalacetic acid	0.01	0.9538	0.23	0.3648	-0.15	0.4835	-0.10	0.4799	0.08	0.5323	0.26	0.2687	-0.11	0.3952	-0.01	0.9481	0.20	0.3141	-0.03	0.8794	0.05	0.7985	0.1052	0.4960
Citric acid	-0.19	0.1140	0.07	0.6130	0.08	0.6088	***-0.32***	***0.0245***	0.16	0.3957	0.29	0.0763	-0.09	0.5394	-0.10	0.5053	0.31	0.0711	0.14	0.4770	-0.01	0.9546	0.2002	0.0737
2-Ketoglutaric acid	-0.25	0.2924	-0.05	0.8130	0.20	0.3805	***-0.65***	***0.0478***	0.24	0.2973	0.19	0.3360	0.00	0.9999	0.27	0.3745	0.18	0.3579	-0.05	0.8094	-0.03	0.8864	0.0166	0.9247
Aconitic acid	***-0.39***	***0.0253***	-0.08	0.7701	-0.33	0.0816	0.24	0.3896	0.27	0.2167	***0.64***	***0.0111***	-0.01	0.9670	0.07	0.8046	***0.41***	***0.0164***	0.31	0.2941	0.36	0.2072	0.0761	0.7606
Fumaric acid	0.00	0.9904	-0.13	0.6249	-0.30	0.2898	-0.18	0.3672	0.55	0.1884	0.19	0.4763	-0.25	0.4478	0.35	0.2531	0.19	0.5000	0.17	0.4959	-0.03	0.9214	-0.0044	0.9808
Malic acid	0.00	0.9931	-0.13	0.7093	-0.34	0.3463	-0.57	0.1233	0.79	0.2228	0.39	0.2850	-0.31	0.4361	0.59	0.3359	0.28	0.4690	0.29	0.4341	-0.03	0.9299	-0.2605	0.2566
Succinic acid	-0.77	0.0567	-0.14	0.7920	-0.52	0.1398	-0.58	0.1586	1.05	0.3261	0.33	0.5734	-0.13	0.8354	-0.20	0.5304	1.09	0.1075	-0.14	0.8067	-0.20	0.6748	0.1439	0.5990
Ribose 5-phosphate	0.41	0.4637	0.79	0.4068	-0.14	0.6259	***-0.47***	***0.0065***	-0.10	0.8303	0.04	0.9029	-0.04	0.8906	0.14	0.3775	-0.59	0.2797	-0.34	0.7140	-0.32	0.1401	0.2708	0.0993
Ribose	1.05	0.3033	0.63	0.4315	-0.22	0.2968	-0.34	0.3167	0.41	0.4582	0.05	0.8874	-0.22	0.3540	0.16	0.6374	-1.38	0.2242	-0.72	0.3779	-0.21	0.3000	0.2160	0.3949
Ribitol	0.11	0.3277	***0.35***	***0.0059***	0.33	0.0980	0.68	0.0657	***0.29***	***0.0172***	***0.38***	***0.0295***	***0.44***	***0.0124***	***0.79***	***0.0477***	***0.25***	***0.0247***	-0.06	0.5622	0.25	0.1520	0.2239	0.4993
Gluconic acid	-0.16	0.4745	0.14	0.4681	0.04	0.6540	-0.06	0.6704	***0.59***	***0.0253***	***0.51***	***0.0167***	***0.22***	***0.0206***	-0.09	0.3031	***0.53***	***0.0050***	***0.38***	***0.0290***	0.21	0.0831	0.1769	0.2167
Ribulose	0.24	0.2307	0.14	0.2372	0.10	0.4003	-0.04	0.8037	***0.30***	***0.0251***	0.01	0.8988	0.06	0.6112	0.08	0.5876	-0.26	0.2487	-0.26	0.0530	0.02	0.8823	0.0374	0.6854
Acetoacetic acid	0.22	0.6550	0.04	0.8125	0.27	0.2576	0.11	0.5476	0.38	0.4779	0.43	0.1112	***0.50***	***0.0358***	0.13	0.4057	-0.16	0.7430	0.04	0.8200	0.56	0.1395	0.2106	0.1477
3-Hydroxybutyric acid	-0.21	0.5008	-0.08	0.8161	-0.02	0.9060	0.06	0.8947	1.13	0.1027	***0.76***	***0.0486***	0.67	0.2457	0.02	0.9559	***0.99***	***0.0417***	0.41	0.2821	0.82	0.1171	-0.1796	0.4823
Spermidine	0.01	0.9568	0.45	0.1440	-0.25	0.3067	0.08	0.7963	0.44	0.1039	0.60	0.0620	0.23	0.4255	-0.17	0.6390	0.43	0.1259	0.53	0.1035	0.00	0.9940	-0.0622	0.7225
Urea	-0.01	0.9211	0.02	0.5794	-0.05	0.4652	0.03	0.5850	0.05	0.6453	-0.01	0.9124	-0.09	0.3448	-0.07	0.1565	-0.02	0.8409	-0.03	0.6525	-0.13	0.1868	0.0096	0.8465
Creatinine	-0.01	0.9743	-0.05	0.8783	-0.17	0.6173	0.64	0.1016	0.14	0.6884	-0.45	0.1935	0.03	0.9355	0.10	0.8677	0.05	0.8618	0.18	0.5855	-0.05	0.8865	-0.1551	0.6641
Arginine	0.02	0.9003	-0.20	0.1926	-0.05	0.5902	-0.08	0.6153	***-0.53***	***0.0135***	***-0.42***	***0.0153***	***-0.33***	***0.0251***	-0.09	0.6066	***-0.50***	***0.0066***	***-0.44***	***0.0342***	***-0.47***	***0.0038***	-0.0415	0.7124
Spermine	0.40	0.0583	***0.52***	***0.0111***	0.38	0.3746	0.25	0.6381	***0.61***	***0.0027***	0.15	0.4836	0.64	0.2330	0.97	0.1436	0.14	0.4532	-0.08	0.4538	0.57	0.3358	0.0903	0.8233
4-Aminobutyric acid	-0.20	0.3930	0.15	0.6211	***1.13***	***0.0451***	-0.05	0.9065	0.35	0.2684	0.13	0.7194	0.20	0.3998	-0.47	0.2843	0.22	0.2885	-0.01	0.9809	-0.60	0.1760	0.1398	0.7357
Putrescine	-0.23	0.0966	0.13	0.4147	0.11	0.4992	-0.02	0.9064	***0.32***	***0.0089***	0.16	0.4561	-0.29	0.1286	0.68	0.3736	***0.46***	***0.0188***	0.01	0.8973	-0.03	0.8369	0.0721	0.6597
Ornithine	0.39	0.1135	0.09	0.6845	-0.13	0.1742	0.04	0.7706	-0.06	0.8185	-0.22	0.1287	-0.10	0.4422	0.07	0.6166	***-0.66***	***0.0449***	***-0.78***	***0.0236***	***-0.26***	***0.0401***	0.0905	0.3575
Glutamic acid	-0.30	0.2853	-0.14	0.5464	-0.38	0.1013	-0.08	0.5118	0.15	0.7026	0.10	0.6729	-0.17	0.5685	0.23	0.3611	0.26	0.3598	-0.20	0.4961	0.03	0.9120	0.0099	0.9225
Homocysteine	***0.35***	***0.0038***	0.09	0.4083	-0.18	0.4608	-0.06	0.7250	-0.01	0.8773	-0.15	0.2086	-0.25	0.0990	0.17	0.2460	***-0.45***	***0.0010***	***-0.56***	***0.0059***	-0.16	0.5330	0.2421	0.1787
Cysteine	-0.03	0.4795	0.02	0.8969	-0.15	0.2719	-0.08	0.5664	0.04	0.5372	0.07	0.5587	-0.15	0.1883	-0.13	0.3433	-0.08	0.4903	0.28	0.4815	-0.06	0.6760	-0.0506	0.5838
Methionine	0.06	0.3431	0.00	0.9491	0.13	0.1236	0.28	0.0613	***-0.36***	***0.0001***	***-0.23***	***0.0374***	-0.11	0.1459	-0.02	0.8271	***-0.43***	***0.0003***	***-0.50***	***0.0002***	***-0.41***	***0.0015***	-0.1296	0.2906
5-Methoxytryptamine	1.29	0.3905	3.17	0.3520	-0.72	0.1966	-0.90	0.2850	-0.14	0.9150	0.14	0.6132	-0.66	0.2081	0.21	0.7953	-3.19	0.1951	-2.20	0.4101	-0.01	0.9811	0.1377	0.6899
2-Ketoadipic acid	-0.20	0.0585	0.40	0.6037	-0.24	0.4101	-1.11	0.6307	0.16	0.4908	0.67	0.2836	-0.36	0.0687	0.12	0.9407	0.19	0.2603	0.59	0.4096	0.36	0.1987	-0.5871	0.7303
Quinolinic acid	-0.10	0.5036	0.22	0.2779	0.30	0.4097	***-0.62***	***0.0185***	-0.19	0.1783	0.18	0.3634	-0.29	0.3132	-0.03	0.8778	-0.10	0.6487	-0.25	0.1826	-0.58	0.1748	0.0072	0.9701
Anthranilic acid	0.41	0.1529	0.12	0.7154	0.08	0.7952	-0.30	0.2532	-0.38	0.3290	-0.10	0.7367	-0.33	0.4711	0.02	0.9639	***-1.05***	***0.0005***	-0.56	0.0691	-0.59	0.0609	-0.0104	0.9421
Kynurenine	-0.05	0.6947	0.00	0.9903	0.27	0.1117	0.13	0.4258	0.07	0.6489	0.21	0.0699	0.16	0.2448	0.23	0.1977	0.00	0.9755	-0.04	0.8293	-0.23	0.2583	0.3128	0.0627
Tryptophan	-0.03	0.8160	-0.03	0.6177	-0.09	0.5838	-0.04	0.4214	-0.09	0.4685	-0.13	0.0528	-0.15	0.1385	0.01	0.9122	-0.10	0.2757	***-0.29***	***0.0028***	-0.10	0.5527	-0.0133	0.7668
Kynurenic acid	***-0.27***	***0.0446***	-0.12	0.5582	-0.11	0.6804	-0.08	0.7428	0.40	0.2715	0.01	0.9490	0.01	0.9723	0.50	0.2970	0.42	0.0786	0.06	0.7796	-0.30	0.3446	0.1516	0.5436
Indol-3-acetic acid	-0.06	0.7606	-0.18	0.1753	***-0.59***	***0.0143***	-0.32	0.0876	-0.37	0.1302	-0.37	0.0918	-0.30	0.2390	-0.02	0.8969	-0.26	0.1018	-0.31	0.0957	-0.26	0.2333	0.1512	0.0557
Dopamine	0.65	0.1404	0.11	0.6066	-0.14	0.1593	0.05	0.7059	-0.02	0.9580	-0.20	0.1489	-0.08	0.5270	0.08	0.5522	-0.86	0.0928	***-0.80***	***0.0232***	***-0.26***	***0.0446***	0.0938	0.3272
Tyrosine	0.03	0.8297	***-0.38***	***0.0043***	-0.09	0.4102	-0.04	0.6836	***-0.35***	***0.0423***	***-0.43***	***0.0229***	-0.18	0.2076	0.03	0.7778	***-0.42***	***0.0051***	-0.24	0.0522	-0.03	0.8361	0.0506	0.5443
Norepinephrine	-0.28	0.6893	-0.79	0.3907	-0.75	0.1649	-2.18	0.3480	0.94	0.2492	0.03	0.9707	0.29	0.7394	-2.32	0.3371	***1.42***	***0.0420***	1.41	0.2083	0.31	0.7175	-0.0831	0.8621
Cystine	0.11	0.5684	0.08	0.5942	0.01	0.9672	-0.21	0.2746	-0.17	0.2169	0.04	0.6742	0.00	0.9832	-0.02	0.9266	-0.40	0.0979	-0.06	0.7229	0.05	0.7395	-0.0771	0.5064
Glycine	-0.03	0.5712	-0.01	0.9323	-0.02	0.7810	***-0.30***	***0.0003***	-0.06	0.5377	-0.10	0.4002	-0.14	0.1108	-0.06	0.2978	***-0.12***	***0.0404***	***-0.42***	***0.0097***	-0.02	0.7957	0.0873	0.1254
Proline	0.01	0.9418	-0.07	0.5422	-0.05	0.4144	-0.02	0.8326	-0.07	0.5627	-0.11	0.2571	0.03	0.7819	-0.03	0.6551	-0.15	0.0574	***-0.56***	***0.0055***	-0.03	0.4600	0.0435	0.5088
Serine	0.00	0.9529	-0.08	0.4503	0.02	0.8455	0.04	0.5189	-0.19	0.0843	-0.24	0.0783	-0.06	0.3724	-0.02	0.8042	***-0.26***	***0.0034***	***-0.53***	***0.0016***	-0.12	0.1583	-0.0282	0.6349
Alanine	-0.03	0.6714	-0.08	0.4238	0.06	0.3795	-0.02	0.7298	-0.09	0.3622	-0.05	0.6551	-0.10	0.2300	0.05	0.4726	-0.15	0.0767	-0.19	0.1515	-0.12	0.0618	-0.0797	0.1469
4-Hydroxyproline	-0.04	0.2367	-0.10	0.1202	0.02	0.7987	***-0.30***	***0.0044***	***-0.26***	***0.0001***	-0.11	0.1238	0.01	0.9402	-0.04	0.7691	-0.07	0.5933	-0.07	0.3786	-0.06	0.6446	0.0388	0.5988
Glutamine	-0.07	0.2109	-0.13	0.2390	0.15	0.4117	0.03	0.8281	***-0.26***	***0.0001***	-0.20	0.1151	0.14	0.3735	0.05	0.3954	***-0.39***	***0.0041***	-0.25	0.0915	-0.14	0.3740	0.1857	0.1586
Aspartic acid	0.14	0.4274	-0.15	0.4737	***-0.23***	***0.0374***	-0.17	0.2607	0.20	0.4543	-0.23	0.2890	-0.10	0.5508	0.07	0.6535	-0.20	0.3045	***-0.33***	***0.0368***	0.07	0.6162	-0.0604	0.3306
Asparagine	-0.02	0.8727	-0.19	0.1850	-0.15	0.4008	-0.18	0.2246	***-0.46***	***0.0012***	-0.28	0.1048	-0.16	0.2314	-0.17	0.2858	***-0.60***	***0.0011***	***-0.72***	***0.0057***	-0.36	0.0986	-0.0637	0.5169
Valine	0.02	0.7138	-0.05	0.6292	0.08	0.3239	-0.15	0.0713	***-0.25***	***0.0079***	***-0.35***	***0.0349***	***-0.39***	***0.0064***	-0.08	0.2592	***-0.43***	***0.00004***	***-0.70***	***0.0009***	***-0.40***	***0.0080***	0.0251	0.7261
Leucine	0.04	0.5827	-0.10	0.3735	0.03	0.5967	-0.03	0.6878	-0.20	0.0692	***-0.35***	***0.0339***	-0.20	0.1020	-0.05	0.3791	***-0.35***	***0.0010***	***-0.67***	***0.0026***	***-0.23***	***0.0466***	0.0336	0.6706
Isoleucine	0.04	0.5607	-0.13	0.2496	0.01	0.9204	-0.18	0.0763	***-0.25***	***0.0316***	***-0.38***	***0.0282***	***-0.31***	***0.0193***	-0.05	0.5451	***-0.42***	***0.0002***	***-0.65***	***0.0023***	***-0.36***	***0.0113***	0.0444	0.5304
Threonine	0.06	0.3589	0.07	0.6175	0.03	0.8018	0.02	0.8342	***-0.44***	***0.0014***	-0.23	0.0505	-0.28	0.0740	-0.18	0.1698	***-0.45***	***0.0011***	***-0.70***	***0.0032***	***-0.40***	***0.0318***	***-0.1890***	***0.0247***
Phenylalanine	0.02	0.7762	-0.02	0.8221	0.20	0.0695	***0.30***	***0.0143***	-0.17	0.1399	-0.09	0.4222	-0.03	0.7372	0.04	0.6388	***-0.26***	***0.0105***	***-0.36***	***0.0021***	***-0.30***	***0.0423***	-0.0207	0.7779
Histidine	0.06	0.3980	-0.14	0.3936	0.14	0.6788	0.01	0.9519	***-0.39***	***0.0018***	-0.30	0.0961	0.02	0.9269	0.03	0.8313	***-0.62***	***0.0004***	-0.38	0.0635	-0.05	0.8747	-0.0160	0.8703
Lysine	0.12	0.2999	-0.24	0.1027	-0.17	0.3721	-0.04	0.8045	***-0.32***	***0.0087***	-0.34	0.0739	0.03	0.8212	-0.07	0.7232	***-0.50***	***0.0049***	***-0.33***	***0.0373***	-0.11	0.5837	***-0.2341***	***0.0280***
Adenosine	***0.86***	***0.0444***	0.46	0.5576	1.24	0.3760	0.09	0.8816	0.38	0.1651	0.06	0.9200	1.96	0.3725	-0.58	0.4588	-0.23	0.4681	-0.97	0.1999	0.35	0.7491	0.0876	0.8873
Homogentisic acid	-0.05	0.8898	0.24	0.5862	0.10	0.7253	0.75	0.1760	0.60	0.4397	-0.05	0.8912	0.23	0.5937	0.79	0.2748	0.68	0.0792	-0.76	0.1144	-0.18	0.6204	0.0612	0.9055
Lactic acid	0.14	0.2466	0.15	0.4441	-0.05	0.6853	-0.04	0.8327	0.41	0.1102	0.22	0.3507	-0.15	0.3829	0.46	0.2391	0.11	0.3608	0.17	0.4885	0.02	0.8568	-0.0452	0.7860

Significantly changed metabolites at P < 0.05 were shown as bold and italic.

Supplementary Table 4 Fold changes and p values for lipid mediators in poly I:C and/or maoto-treated rats

		P/C				M/C				PM/P			
		2h		4h		2h		4h		2h		4h	
**Compound name**	**Category**	log_2FC	*p* value	log_2FC	*p* value	log_2FC	*p* value	log_2FC	*p* value	log_2FC	*p* value	log_2FC	*p* value
13-HpODE	LA	0.75	0.6531	0.25	0.8503	2.37	0.3994	-2.47	0.2631	-0.76	0.6059	-1.63	0.3401
9-HpODE	LA	0.65	0.6394	0.35	0.7895	2.26	0.3818	-2.31	0.2671	-0.77	0.5599	-1.51	0.3475
12,13-DiHOME	LA	0.25	0.2970	0.12	0.7347	***0.57***	***0.0009***	-0.31	0.3719	0.26	0.2751	-0.28	0.2750
9,10-DiHOME	LA	0.31	0.1033	-0.14	0.7070	***0.50***	***0.0013***	-0.34	0.3572	0.19	0.3309	-0.10	0.6925
13-KODE	LA	-0.08	0.8227	0.50	0.6301	0.39	0.6113	-1.68	0.2244	-0.15	0.6568	-1.45	0.2447
12,13-EpOME	LA	0.34	0.2900	0.33	0.5477	0.34	0.2241	-0.45	0.3320	-0.42	0.2139	-0.60	0.2897
13-HODE	LA	0.15	0.3700	0.36	0.4425	0.14	0.3307	-0.46	0.2859	-0.11	0.5325	-0.80	0.1001
9,10-EpOME	LA	0.10	0.7483	0.36	0.5162	0.04	0.9043	-0.80	0.1154	-0.30	0.4440	-1.14	0.0991
9-HODE	LA	-0.02	0.8764	0.50	0.3400	-0.05	0.7651	-0.53	0.2103	-0.13	0.4561	-0.97	0.1031
9-KODE	LA	-0.17	0.5385	0.97	0.2862	-0.09	0.7688	-0.95	0.0823	-0.03	0.9170	-1.16	0.2474
15-HEDE	EDA	-0.05	0.8310	0.71	0.1973	0.33	0.2553	-0.76	0.1497	0.14	0.6492	-1.18	0.0592
15-KEDE	EDA	-0.26	0.3891	0.83	0.1917	0.13	0.6161	-0.62	0.1001	0.21	0.4477	-1.15	0.1024
15-HETrE	DGLA	0.20	0.4262	1.11	0.1346	***0.77***	***0.0106***	0.00	0.9928	0.25	0.3707	-0.98	0.1585
13,14-dihydro-15-keto-tetranor-PGE2	AA	***2.37***	***0.0113***	***2.09***	***0.0092***	0.05	0.9375	-0.80	0.1002	-0.40	0.4569	***-2.03***	***0.0098***
N-acetyl-LTE4	AA	***2.21***	***0.0298***	1.28	0.1276	-1.85	0.1154	-1.41	0.1760	***-2.45***	***0.0250***	-0.96	0.2437
6,15-diketo-13,14-dihydro-PGF1α	AA	***1.63***	***0.0071***			0.33	0.5333			-0.52	0.2125		
6-keto-PGF1α	AA	***1.21***	***0.0027***	0.67	0.0545	0.16	0.3939	0.08	0.6159	-0.35	0.2407	0.01	0.9842
tetranor-PGDM	AA	***1.08***	***0.0003***	***1.52***	***0.0005***	-0.07	0.8307	-0.07	0.7573	***-0.55***	***0.0433***	***-1.14***	***0.0016***
LTE4	AA	***1.04***	***0.0282***	0.58	0.0801	***-0.95***	***0.0058***	***-1.00***	***0.0439***	***-0.91***	***0.0490***	-0.51	0.0661
13,14-dihydro-15-keto-PGE2	AA	***1.01***	***0.0023***	0.22	0.4496	0.22	0.3285	0.13	0.7144	-0.04	0.8872	0.42	0.1718
13,14-dihydro-15-keto-tetranor-PGF1β	AA	***0.93***	***0.0049***	0.27	0.5503	0.11	0.6942	0.40	0.3682	0.11	0.7071	-0.01	0.9826
TXB2	AA	0.70	0.4221	0.28	0.6194	1.36	0.3144	-0.87	0.2841	0.92	0.3859	-0.46	0.0980
13,14-dihydro-15-keto-PGD2	AA	0.70	0.1162			-0.06	0.8652			-0.34	0.4100		
19-HETE	AA	0.66	0.0962	-0.33	0.6192	***1.44***	***0.0003***	0.46	0.4879	0.46	0.0818	0.72	0.3451
PGE2	AA	0.59	0.0568	0.59	0.1483	-0.31	0.3963	0.22	0.5937	-0.15	0.6300	0.22	0.5428
17-HETE	AA	0.59	0.0803	0.13	0.6336	0.20	0.6332	0.07	0.8047	0.42	0.1359	0.16	0.5173
13,14-dihydro-15-keto-PGF2α	AA	***0.56***	***0.0142***	-0.30	0.6206	0.41	0.1247	-0.29	0.6132	0.05	0.8054	0.38	0.3757
5,6-DHET-lactone	AA	0.53	0.2382			-0.43	0.4764			-0.87	0.0598		
12-HHT	AA	0.33	0.4181	1.13	0.1147	0.48	0.3310	-0.39	0.4209	-0.01	0.9898	-1.45	0.0696
PGD2	AA	0.28	0.2951	0.92	0.1679	-0.26	0.4960	-0.58	0.2525	-0.11	0.7433	-1.25	0.0883
18-HETE	AA	0.25	0.2581	-0.05	0.8496	0.16	0.3714	-0.08	0.7780	-0.26	0.2852	-0.07	0.6116
PGF2α	AA	0.24	0.1821	-0.04	0.6995	0.30	0.2198	-0.20	0.0578	0.14	0.5203	-0.13	0.1732
11-HETE	AA	0.23	0.1620	0.38	0.3578	0.15	0.5392	-0.41	0.1778	0.04	0.8343	-0.66	0.1386
16-HETE	AA	0.13	0.5505	0.70	0.1214	0.13	0.4557	-0.23	0.4603	-0.02	0.9244	-0.94	0.0511
AA	AA	0.12	0.2034	0.14	0.3745	0.19	0.0823	-0.02	0.9304	-0.10	0.2478	-0.04	0.7424
14,15-DHET	AA	0.07	0.7143	0.40	0.0640	***0.79***	***0.0186***	0.01	0.9663	0.22	0.2042	***-0.41***	***0.0274***
8,9-DHET	AA	0.07	0.7309	0.42	0.1231	***0.82***	***0.0012***	-0.02	0.9249	0.16	0.4636	***-0.59***	***0.0388***
20-carboxy-AA	AA	0.03	0.7966	0.32	0.2132	***0.40***	***0.0360***	0.17	0.3808	***0.28***	***0.0332***	0.00	0.9975
5-HETE	AA	-0.07	0.6520	0.39	0.3962	-0.06	0.7039	-0.47	0.1829	0.01	0.9756	-0.73	0.1454
15-HETE	AA	-0.12	0.5541	0.56	0.3217	-0.07	0.7419	-0.49	0.2419	-0.02	0.9338	-0.78	0.1921
8-HETE	AA	-0.12	0.5009	0.39	0.3355	0.09	0.6480	-0.40	0.2308	0.05	0.7846	-0.62	0.1379
8-iso-PGE2	AA	-0.13	0.5473	0.77	0.2160	-0.45	0.0682	-0.43	0.3110	-0.23	0.2909	-0.98	0.1384
5,6-DHET	AA	-0.14	0.5072	0.11	0.7039	***0.42***	***0.0260***	***-0.54***	***0.0420***	0.23	0.2591	***-0.71***	***0.0484***
11,12-DHET	AA	-0.15	0.5028	0.23	0.2504	0.90	0.0627	0.13	0.6332	0.19	0.2896	-0.31	0.0603
15-keto-PGE2	AA	-0.15	0.6629	0.64	0.3920	-0.28	0.4705	-0.61	0.2815	-0.07	0.8620	-1.08	0.1982
8-iso-15-keto-PGF2α	AA	-0.16	0.6767	1.04	0.1252	-0.41	0.2673	-0.68	0.2140	-0.14	0.7150	-0.99	0.1440
5-KETE	AA	-0.18	0.4710	0.36	0.3886	-0.29	0.2265	-0.45	0.2536	-0.02	0.9210	-0.67	0.1416
11,12-EET	AA	-0.22	0.3565	0.37	0.3502	***-0.50***	***0.0258***	-0.33	0.3211	-0.13	0.6213	-0.59	0.1678
9-HETE	AA	-0.22	0.4845	0.63	0.2478	0.09	0.8480	-0.42	0.2675	0.30	0.4276	-1.09	0.0926
20-HETE	AA	-0.23	0.4801	0.52	0.1489	0.51	0.1962	-0.20	0.5039	***0.70***	***0.0100***	0.06	0.8625
5,6-EET	AA	-0.26	0.0735	0.11	0.7312	-0.22	0.2253	-0.49	0.1675	0.12	0.5228	-0.56	0.1479
8,9-EET	AA	-0.32	0.3709	0.06	0.8952	-0.23	0.5083	-0.33	0.4300	0.27	0.5735	-0.66	0.1796
14,15-EET	AA	-0.40	0.1880	0.01	0.9899	-0.23	0.5180	-0.59	0.2497	0.02	0.9587	-0.47	0.4056
12-KETE	AA	-0.45	0.3411	0.53	0.5619	0.74	0.2589	-1.34	0.1886	0.54	0.3234	-1.45	0.1940
12-HETE	AA	-0.61	0.6200	0.37	0.2393	1.25	0.3376	-0.38	0.2834	1.33	0.3033	-0.36	0.1718
5-iPF2α-VI	AA	-0.73	0.0916	0.38	0.2194	-0.16	0.6688	-0.25	0.4674	***0.75***	***0.0312***	-0.41	0.2011
13,14-dihydro-15-keto-tetranor-PGF1α	AA			0.18	0.5252			0.41	0.2744			0.16	0.5230
1a1b-dihomo-PGF2α	ADA			0.80	0.3529			***2.49***	***0.0001***			1.56	0.0930
13-HOTrE	ALA	0.16	0.3917	0.11	0.8375	0.23	0.1541	-0.43	0.4332	-0.20	0.3117	-0.60	0.2371
9-HOTrE	ALA	0.04	0.8999	0.61	0.3888	0.03	0.9263	-0.77	0.2123	-0.05	0.8295	-1.19	0.1378
12-HEPE	EPA	-0.06	0.9238	0.02	0.9552	1.09	0.2369	-0.37	0.2824	0.67	0.4123	-0.27	0.2331
17,18-DiHETE	EPA	0.19	0.2825	0.32	0.2658	***0.60***	***0.0047***	-0.17	0.5911	-0.30	0.1185	-0.38	0.0834
EPA	EPA	0.28	0.0660	-0.05	0.8465	***0.56***	***0.0003***	-0.10	0.7381	0.04	0.7844	0.03	0.8922
15-HEPE	EPA	0.28	0.2045	0.06	0.8874	***0.47***	***0.0273***	-0.29	0.5127	-0.02	0.9196	-0.55	0.0617
14,15-DiHETE	EPA	0.14	0.4729	0.00	0.9926	***0.41***	***0.0285***	-0.20	0.6575	-0.13	0.5713	-0.17	0.4546
5-HEPE	EPA	0.16	0.4369	0.04	0.8958	***0.39***	***0.0390***	-0.35	0.3518	0.10	0.6496	-0.47	0.0748
18-HEPE	EPA	0.02	0.9192	0.07	0.9198	0.08	0.6537	-0.59	0.4113	-0.16	0.4555	-0.77	0.2472
5,6-DiHETE	EPA	-0.19	0.3168	0.03	0.9096	-0.03	0.8856	-0.23	0.4434	***-0.44***	***0.0402***	-0.44	0.1635
PGD3	EPA			-0.03	0.9637			-0.49	0.3935			-0.55	0.4377
14-HDoHE	DHA	-0.32	0.5537	0.40	0.3065	0.82	0.2414	-0.39	0.3216	0.58	0.3360	-0.58	0.1081
DHA	DHA	0.22	0.1171	0.09	0.6708	***0.46***	***0.0015***	-0.10	0.6829	0.05	0.7165	0.03	0.8500
11-HDoHE	DHA	0.05	0.8270	0.13	0.7422	0.35	0.1193	-0.42	0.2230	0.03	0.8770	-0.58	0.1971
17-HDoHE	DHA	-0.12	0.6426	0.47	0.3175	0.30	0.3432	-0.62	0.1899	0.18	0.4915	-0.79	0.0975
10-HDoHE	DHA	0.01	0.9795	0.14	0.7042	0.15	0.4008	-0.47	0.1814	-0.04	0.8516	-0.60	0.1092
4-HDoHE	DHA	-0.04	0.8549	0.33	0.5302	0.12	0.5110	-0.59	0.1935	0.03	0.8798	-0.79	0.1654
7-HDoHE	DHA	0.02	0.9295	0.23	0.4673	0.11	0.6263	-0.26	0.3965	-0.17	0.4921	-0.39	0.2448
16-HDoHE	DHA	-0.12	0.4789	0.38	0.4556	0.10	0.5803	-0.60	0.2229	0.16	0.3512	-0.76	0.1515
8-HDoHE	DHA	-0.07	0.7429	0.23	0.4166	0.09	0.6202	-0.20	0.4529	-0.15	0.5244	-0.44	0.1105
13-HDoHE	DHA	0.01	0.9751	0.18	0.6626	0.03	0.8458	-0.44	0.2388	-0.06	0.7626	-0.62	0.1707
20-HDoHE	DHA	-0.13	0.4868	0.44	0.4254	-0.10	0.5761	-0.54	0.3037	0.06	0.7400	-0.71	0.2086

Significantly changed metabolites at P < 0.05 were shown as bold and italic.

Supplementary figure 1

A. Comprehensive analysis / B. Profiling

1. Detection of compound and metabolite profiling after administration of maoto

Maoto-derived compounds
- Major compounds in maoto were identified.
    - Ephedrines/Prunasin/Cinnamic acid/Glycyrretinic acid
- Numerous compound peaks were detected in rat plasma.
    - Unchanged compounds in maoto
    - Maoto-derived metabolites

Endogenous compounds
- Several kinds of endogenous metabolites were increased by maoto administration
    - Lysophospholipids/Carnitins

Undetermined compounds

2. Pharmacological profiling of maoto on flu-like symptoms

Phenotype
- Maoto ameliorated the polyI:C-induced decrease in locomotor activity and body weight
- Maoto inhibited polyI:C-induced proinflammatory cytokines and enhanced anti-inflammatory cytokines
    - TNF-α/IL-1β/IL-10/IFN-γ

Metabolomics
- Maoto affected primary metabolites
    - Amino acids/ metabolites of TCA cycle / ketone bodies
- Maoto decreased polyI:C-induced inflammatory lipid mediators
    - Prostaglandins / Leukotrienes
- Maoto affected broad lipid mediators
    - Metabolites of ω3 / ω6 lipid mediators

C. Integrated hypothesis of mode of action

Literature knowledge / database curation

- **Action of compounds detected in rat plasma**
    - Direct effect of major compounds on proinflammatory cytokines
    - Interaction between maoto-derived compounds and CYPs associated with lipid mediator metabolism
    - Unknown function of undetermined compounds
- **Function of maoto increased lysophospholipids**
    - Effect on proinflammatory cytokines
    - Source of lipid mediators; modulated lipid mediator balance
- **Function of maoto increased lipid mediators**
    - Anti-inflammatory effects of ω3 fatty acid metabolites
    - Modulation of metabolic pathway of proinflammatory lipid mediators
- **Changes of primary metabolites by maoto administration**
    - Effect of maoto on energy metabolism

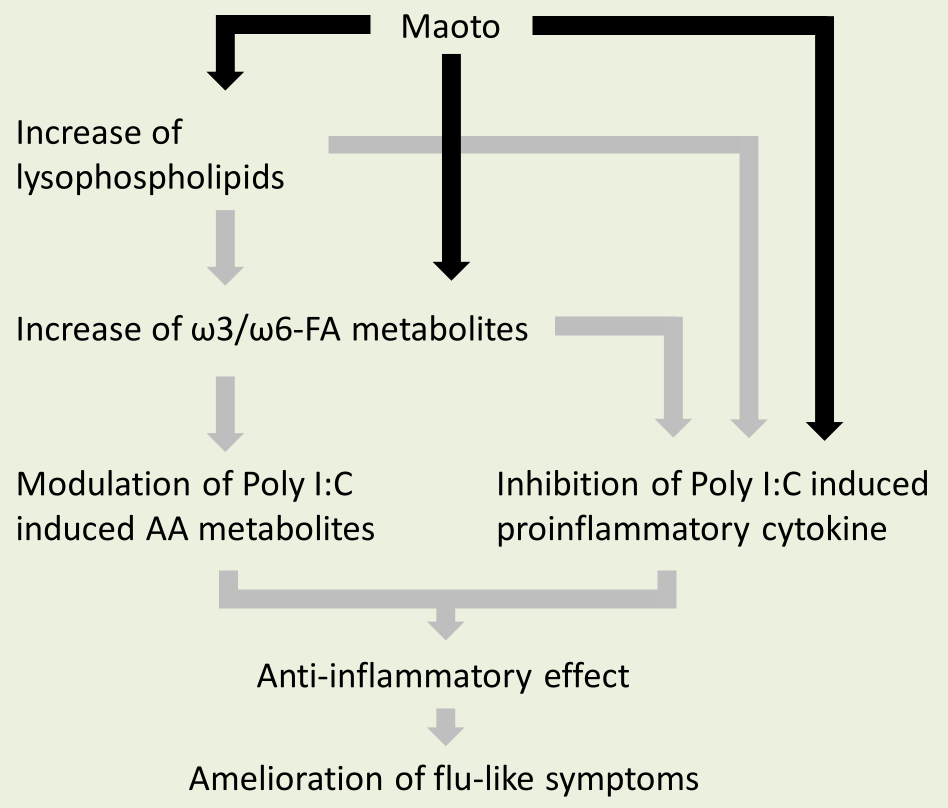

Supplementary Figure 2a

Category	Plasma (h) 1	8	CCD (Formula)	tR (min)	Measured Mass	Theoretical Mass	Ionization	Error (ppm)	Identification	MS$_2$ fragment ions
Maoto original compounds			C8H7N1O1	3.80	134.0601	134.0600	[M + H]+	0.29	Mandelonitrile	ND
			C9H11N1	3.76	134.0965	134.0964	[M + H]+	0.19	-	ND
			C10H16	6.28	137.1325	137.1325	[M + H]+	0.22	-	81 (1000), 95 (148), 146 (111)
			C7H7N1O2	0.94	138.0550	138.0550	[M + H]+	0.04	-	ND
			C7H13N1O2	1.31	144.1019	144.1019	[M + H]+	0.15	Stachydrine	ND
			C10H13N1	4.49	148.1121	148.1121	[M + H]+	0.14	5,6,7,8-Tetrahydrolepidine	ND
			C8H9N1O2	3.88	152.0707	152.0706	[M + H]+	0.31	-	ND
			C9H13N1O1	3.76	152.1070	152.1070	[M + H]+	0.25	Norephedrine	134 (1000), 134 (129)
			C9H14O2	7.88	155.1067	155.1067	[M + H]+	0.28	-	ND
			C6H9N3O2	0.96	156.0768	156.0768	[M + H]+	0.09	Histidine	ND
			C4H6N4O3	0.93	159.0513	159.0513	[M + H]+	0.13	Allantoin	ND
			C6H10O5	0.90	163.0601	163.0601	[M + H]+	0.16	-	ND
			C10H14N2	1.21	163.1230	163.1230	[M + H]+	0.14	-	ND
			C10H15N1O1	4.47	166.1227	166.1226	[M + H]+	0.30	Ephedrine, Pseudoephedrine	148 (1000), 148 (143)
			C11H17N1O1	4.89	180.1383	180.1383	[M + H]+	0.23	Methylephedrine	162 (1000)
			C13N2O1	5.06	201.0083	201.0083	[M + H]+	-0.21	-	ND
			C12H8O3	8.81	201.0546	201.0546	[M + H]+	0.06	-	ND
			C15H12O4	7.27	257.0808	257.0808	[M + H]+	-0.19	Liquiritigenin	ND
			C19H30O10	23.79	419.1910	419.1912	[M + H]+	-0.37	-	ND
Maoto-derived metabolites			C9H9N1O3	5.79	180.0656	180.0655	[M + H]+	0.22	Hippuric acid	163 (1000), 162 (444), 163 (333), 105 (222)
			C11H17N1O2	5.59	196.1332	196.1332	[M + H]+	0.06	Methylephedrine N-oxide	ND
			C30H46O4	14.56	471.3468	471.3469	[M + H]+	-0.21	Glycyrrhetinic acid	425 (1000), 235 (842), 263 (737), 453 (684), 189 (579), 407 (526), 426 (526), 217 (474), 454 (421), 191 (368)
Total	15	12	22							

Supplementary Figure 2b

Category		Plasma (h) 1	Plasma (h) 8	CCD (Formula)	tR (min)	Measured Mass	Theoretical Mass	Ionization	Error (ppm)	Identification	MS2 fragment ions
Endogenous metabolites	Metabolites	■	■	C5H4N4O3	0.95	169.0356	169.0356	[M + H]+	0.16	Urate	ND
		■		C22H41N1O2	16.14	352.3210	352.3210	[M + H]+	-0.16	Anandamide	ND
		■	■	C26H43N1O6	9.97	466.3162	466.3163	[M + H]+	-0.18	Glycocholic acid	448 (1000), 430 (696), 374 (51), 373 (43), 412 (21), 431 (19), 449 (18), 355 (16), 458 (16)
	Acyl carnitines	■		C10H19N1O4	1.03	218.1386	218.1387	[M + H]+	-0.24	Propionylcarnitine	ND
		■		C25H45N1O4	12.60	424.3420	424.3421	[M + H]+	-0.22	Linoelaidyl carnitine, Linoleyl carnitine	ND
		■		C25H47N1O4	13.18	426.3577	426.3578	[M + H]+	-0.23	Oleoylcarnitine, Vaccenyl carnitine, Elaidic carnitine, 11Z-Octadecenylcarnitine	ND
		■		C25H49N1O4	13.90	428.3733	428.3734	[M + H]+	-0.30	Stearoylcarnitine	ND
	Phospholipids	■		C21H37O6P1	14.94	417.2400	417.2401	[M + H]+	-0.17	CPA(18:2(9Z,12Z)/0:0)	ND
		■		C21H39O7P1	14.94	435.2506	435.2506	[M + H]+	-0.14	LPA(0:0/18:2(9Z,12Z)), LPA(18:2(9Z,12Z)/0:0)	ND
		■		C21H44N1O6P1	13.64	438.2978	438.2979	[M + H]+	-0.24	PE(P-16:0/0:0)	ND
		■		C21H44N1O7P1	12.99	454.2927	454.2928	[M + H]+	-0.31	PC(13:0/0:0), PE(16:0/0:0), PE(0:0/16:0)	ND
		■		C23H39O7P1	14.80	459.2506	459.2506	[M + H]+	-0.14	PA(20:4(5Z,8Z,11Z,14Z)/0:0)	ND
		■		C23H48N1O6P1	15.19	466.3291	466.3292	[M + H]+	-0.18	PC(P-15:0/0:0), PE(O-18:1(9Z)/0:0), PE(P-18:0/0:0)	ND
		■		C22H46N1O7P1	12.08	468.3084	468.3085	[M + H]+	-0.15	PC(O-12:0/2:0), PC(14:0/0:0), PC(0:0/14:0), PE(17:0/0:0)	ND
		■		C23H44N1O7P1	12.72	478.2928	478.2928	[M + H]+	-0.09	PE(0:0/18:2(9Z,12Z)), PE(18:2(9Z,12Z)/0:0)	ND
		■		C24H48N1O6P1	13.78	478.3291	478.3292	[M + H]+	-0.22	PE(P-19:1(12Z)/0:0)	ND
		■		C23H46N1O7P1	13.52	480.3084	480.3085	[M + H]+	-0.11	PE(0:0/18:1(11Z)), PE(0:0/18:1(9Z)), PE(18:1(11Z)/0:0), PE(18:1(9Z)/0:0)	ND
		■		C24H50N1O6P1	14.37	480.3447	480.3449	[M + H]+	-0.23	PC(O-16:1(11Z)/0:0), PC(O-16:1(9E)/0:0), PC(O-16:1(9Z)/0:0), PC(O-16:1(1E)/0:0)	ND
		■	■	C23H48N1O7P1	12.77	482.3240	482.3241	[M + H]+	-0.20	PC(15:0/0:0), PC(14:0/O-1:0), PC(7:0/O-8:0), PE(18:0/0:0), PE(0:0/18:0)	ND
		■		C23H48N1O7P1	14.65	482.3240	482.3241	[M + H]+	-0.20	PC(15:0/0:0), PC(14:0/O-1:0), PC(7:0/O-8:0), PE(18:0/0:0), PE(0:0/18:0)	464 (1000), 341 (398), 421 (19), 465 (14), 310 (8)
		■		C24H52N1O6P1	14.46	482.3604	482.3605	[M + H]+	-0.25	PC(O-8:0/O-8:0), PC(O-16:0/0:0)	341 (1000), 464 (105), 454 (100), 184 (91), 465 (68), 440 (50)
		■		C26H52N1O6P1	15.75	506.3604	506.3605	[M + H]+	-0.22	PC(P-18:1(9Z)/0:0), CerP(d18:1/8:0)	ND
		■		C25H50N1O7P1	13.37	508.3396	508.3398	[M + H]+	-0.28	PC(17:1(10Z)/0:0), PC(17:1(9Z)/0:0), PE(20:1(11Z)/0:0), PE(0:0/20:1(11Z))	ND
		■		C26H54N1O6P1	14.82	508.3760	508.3762	[M + H]+	-0.21	PC(O-18:1(11Z)/0:0), PC(O-18:1(9Z)/0:0), PC(O-18:1(1E)/0:0), PC(P-18:0/0:0)	490 (1000), 367 (556), 490 (444), 184 (333), 449 (333), 499 (333), 431 (278), 481 (278), 470 (167)
		■		C26H48N1O7P1	12.48	518.3241	518.3241	[M + H]+	-0.09	PC(18:3(9Z,12Z,15Z)/0:0), PC(18:3(6Z,9Z,12Z)/0:0)	ND
		■		C26H52N1O7P1	13.88	522.3552	522.3554	[M + H]+	-0.36	PC(18:1(6Z)/0:0), PC(18:1(9E)/0:0), PC(18:1(9Z)/0:0), PC(18:1(11Z)/0:0)	504 (1000), 184 (941), 505 (7), 166 (4), 258 (3), 478 (3), 185 (1), 340 (1), 513 (1)
		■	■	C26H54N1O7P1	15.77	524.3710	524.3711	[M + H]+	-0.21	PC(18:0/0:0)	506 (1000), 184 (229), 507 (49), 258 (5), 185 (3), 341 (2), 508 (2), 166 (1), 185 (1), 346 (1)
		■		C27H54N1O7P1	14.99	536.3710	536.3711	[M + H]+	-0.18	PC(O-16:0/3:1(2E)), PC(19:1(9Z)/0:0), PE(22:1(11Z)/0:0), PE(0:0/22:1(13Z)), PE(22:1(13Z)/0:0)	ND
		■		C27H56N1O7P1	16.97	538.3866	538.3867	[M + H]+	-0.21	PC(19:0/0:0)	520 (1000), 184 (294), 521 (271), 185 (28)
		■		C27H56N1O7P1	23.02	538.3867	538.3867	[M + H]+	-0.08	PC(O-16:0/3:0), PC(O-17:0/2:0), PC(O-18:0/1:0), PC(19:0/0:0), PE(22:0/0:0), PE(0:0/22:0)	ND
		■		C26H50N1O7P1	13.15	542.3216	542.3217	[M + Na]+	-0.28	PC(18:2(2E,4E)/0:0), PC(18:2(9Z,12Z)/0:0)	ND
		■		C28H54N1O7P1	14.61	548.3710	548.3711	[M + H]+	-0.19	PC(20:2(11Z,14Z)/0:0)	530 (1000), 184 (175), 531 (20), 542 (15), 543 (6), 258 (5), 501 (2), 519 (2), 527 (2), 536 (2)
		■		C28H56N1O7P1	16.12	550.3866	550.3867	[M + H]+	-0.19	PC(20:1(9Z)/0:0), PC(20:1(11Z)/0:0)	532 (1000), 184 (181), 533 (25), 545 (7), 258 (3), 540 (2)
		■		C30H54N1O7P1	14.23	572.3709	572.3711	[M + H]+	-0.23	PC(22:4(7Z,10Z,13Z,16Z)/0:0)	ND
			■	C30H62N1O7P1	16.19	580.4336	580.4337	[M + H]+	-0.04	PC(O-16:0/6:0), PC(O-18:0/4:0), PC(O-20:0/2:0), PC(22:0/0:0)	ND
Total		34	5	35							

Supplementary Figure 2c

Category	Plasma (hr) 1	8	CCD (Formula)	tR (min)	Measured Mass	Theoretical Mass	Ionization	Error (ppm)
Undetermined compounds	■		C6H15N1	14.39	102.1277	102.1277	[M + H]+	0.16
	■		C6H15N1	18.92	102.1277	102.1277	[M + H]+	0.20
	■		C5H13N1O1	13.13	104.1070	104.1070	[M + H]+	0.00
	■		C5H13N1O1	15.76	104.1070	104.1070	[M + H]+	0.09
	■		C7H4O1	5.79	105.0335	105.0335	[M + H]+	0.21
	■		C5H9N1O2	7.22	116.0706	116.0706	[M + H]+	0.30
		■	C8H11N1	12.40	122.0964	122.0964	[M + H]+	0.16
		■	C10H16	7.14	137.1325	137.1325	[M + H]+	0.18
		■	C10H16	5.29	137.1325	137.1325	[M + H]+	0.22
	■		C9H14O1	3.56	139.1118	139.1117	[M + H]+	0.33
	■	■	C8H14O2	4.69	143.1067	143.1067	[M + H]+	0.27
	■		C8H6O3	1.12	151.0390	151.0390	[M + H]+	0.36
	■		C6H14O4	10.01	151.0965	151.0965	[M + H]+	0.23
		■	C6H14O4	5.44	151.0965	151.0965	[M + H]+	0.23
	■		C3H8N2O3S1	18.16	153.0328	153.0328	[M + H]+	-0.13
	■		C7H11N3O2	0.86	170.0924	170.0924	[M + H]+	0.17
	■		C12H11N1	13.40	170.0965	170.0964	[M + H]+	0.22
		■	C4H12O3S2	12.54	173.0302	173.0301	[M + H]+	0.70
		■	C7H5N1O1S2	23.13	183.9885	183.9885	[M + H]+	-0.24
	■	■	C2H1Cl5	0.86	200.8592	200.8594	[M + H]+	-0.79
		■	C6H10N2S3	1.30	207.0080	207.0079	[M + H]+	0.35
	■		C15H22O1	9.26	219.1743	219.1743	[M + H]+	-0.07
		■	C7H9O4P1S1	0.95	221.0032	221.0032	[M + H]+	0.12
	■		C7H13O4P1S1	0.86	225.0345	225.0345	[M + H]+	0.06
		■	C6H2N4S3	0.79	226.9515	226.9514	[M + H]+	0.10
	■		C12H18O4	7.18	227.1278	227.1278	[M + H]+	-0.03
	■		C13H25N1O2	17.39	228.1958	228.1958	[M + H]+	0.17
	■		C12H12N2O3	6.76	233.0921	233.0921	[M + H]+	-0.01
	■		C11H18N6	21.02	235.1664	235.1666	[M + H]+	-0.67
	■		C8H22N6S1	12.15	235.1698	235.1699	[M + H]+	-0.67
		■	C9H20N4O4	0.91	249.1557	249.1557	[M + H]+	-0.11
	■	■	C12H26O5	8.30	251.1853	251.1853	[M + H]+	-0.11
	■		C20H28	16.44	269.2263	269.2264	[M + H]+	-0.13
	■		C8H17N9O1S1	13.16	288.1348	288.1350	[M + H]+	-0.44
	■		C10H21N7O1S1	13.77	288.1602	288.1601	[M + H]+	0.47
	■		C8H19N9O1S1	15.77	290.1505	290.1506	[M + H]+	-0.36
		■	C12H15N1O4S2	6.28	302.0514	302.0515	[M + H]+	-0.28
	■		C8H9N3O6S2	11.14	308.0006	308.0006	[M + H]+	0.03
	■		C12H19N7O3	15.36	310.1621	310.1622	[M + H]+	-0.50
		■	C20H38O1S1	13.54	327.2713	327.2716	[M + H]+	-0.95
	■		C14H24N4O6	0.94	345.1768	345.1769	[M + H]+	-0.24
	■		C24H47N1O1	20.63	366.3730	366.3730	[M + H]+	-0.02
		■	C22H42O4	10.90	371.3156	371.3156	[M + H]+	-0.04
	■		C24H38O4	4.73	391.2842	391.2843	[M + H]+	-0.16
	■		C18H41N5S2	15.10	392.2876	392.2876	[M + H]+	-0.13
		■	C27H42O4	13.66	431.3155	431.3156	[M + H]+	-0.22
	■		C27H42O5	14.70	447.3104	447.3105	[M + H]+	-0.19
		■	C26H41N1O6	9.49	464.3006	464.3007	[M + H]+	-0.14
		■	C20H44N6O2S2	9.49	465.3039	465.3040	[M + H]+	-0.20
	■		C19H53N5O5S2	13.66	496.3559	496.3561	[M + H]+	-0.41
	■		C20H50N4O6S2	13.78	506.3168	506.3172	[M]	-0.73
	■		C33H38N2O9	14.06	607.2649	607.2650	[M + H]+	-0.15
	■		C52H95N1O1	13.09	749.7409	749.7414	[M]	-0.57
	■		C23H11O25P1S2	15.76	781.8774	781.8768	[M]	0.69
	■		C43H62O16	9.61	835.4104	835.4111	[M + H]+	-0.79
	■		C58H98O29	9.70	1259.6257	1259.6267	[M + H]+	-0.77
Total	40	19	56					

Supplementary Figure 3

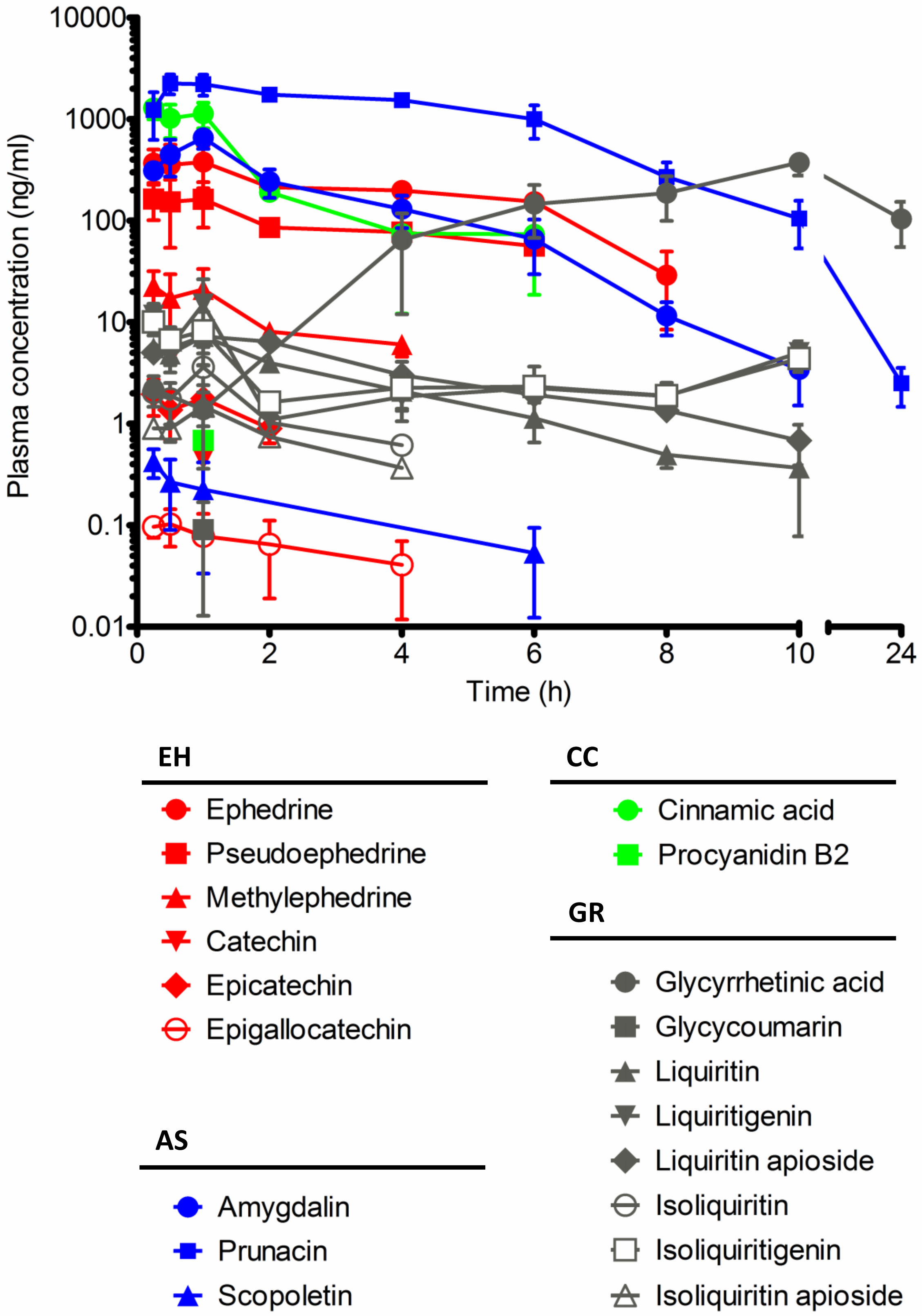

Supplementary Figure 4

Metabolic reactions of detected metabolites

Metabolism in enterobacteria

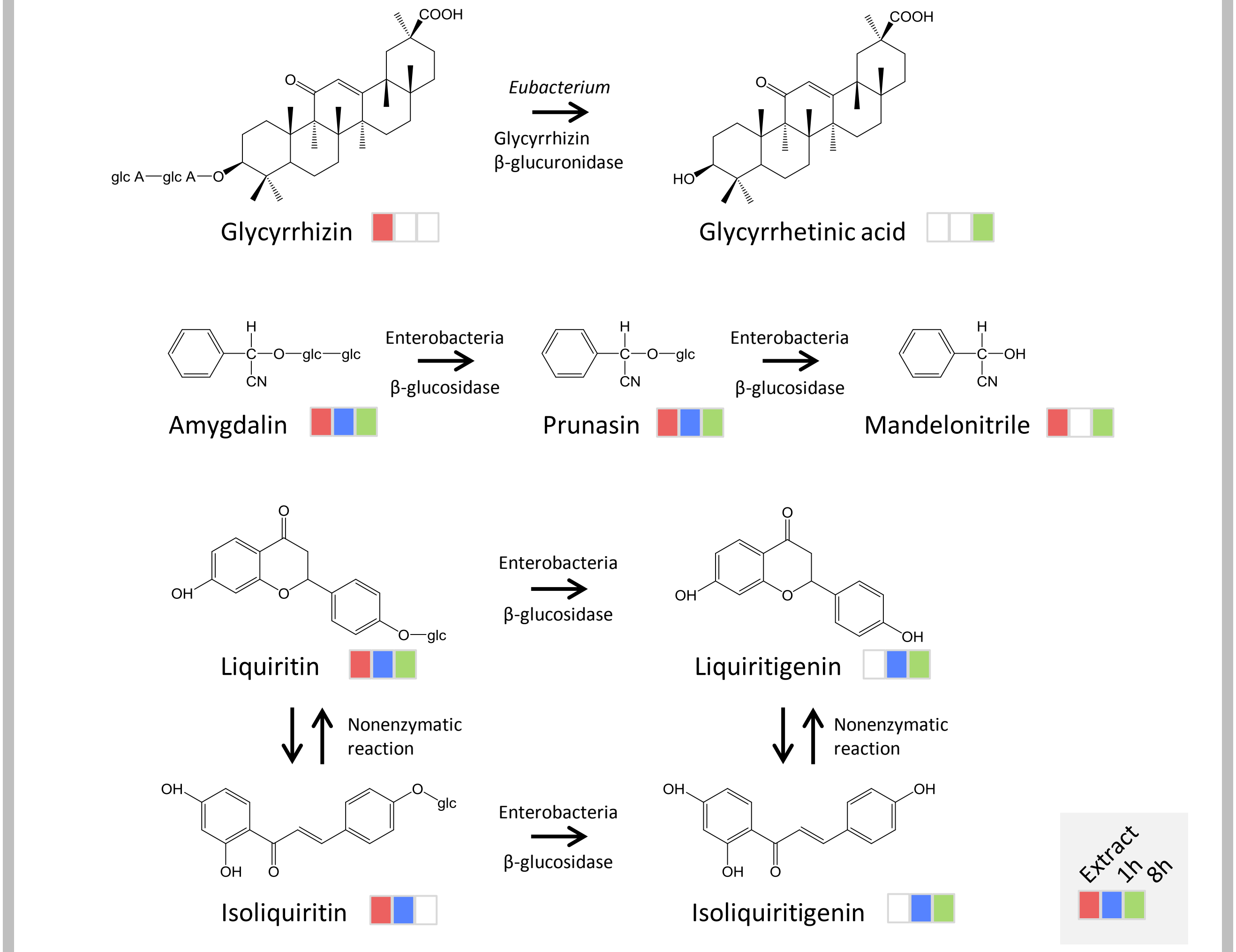

Supplementary Figure5

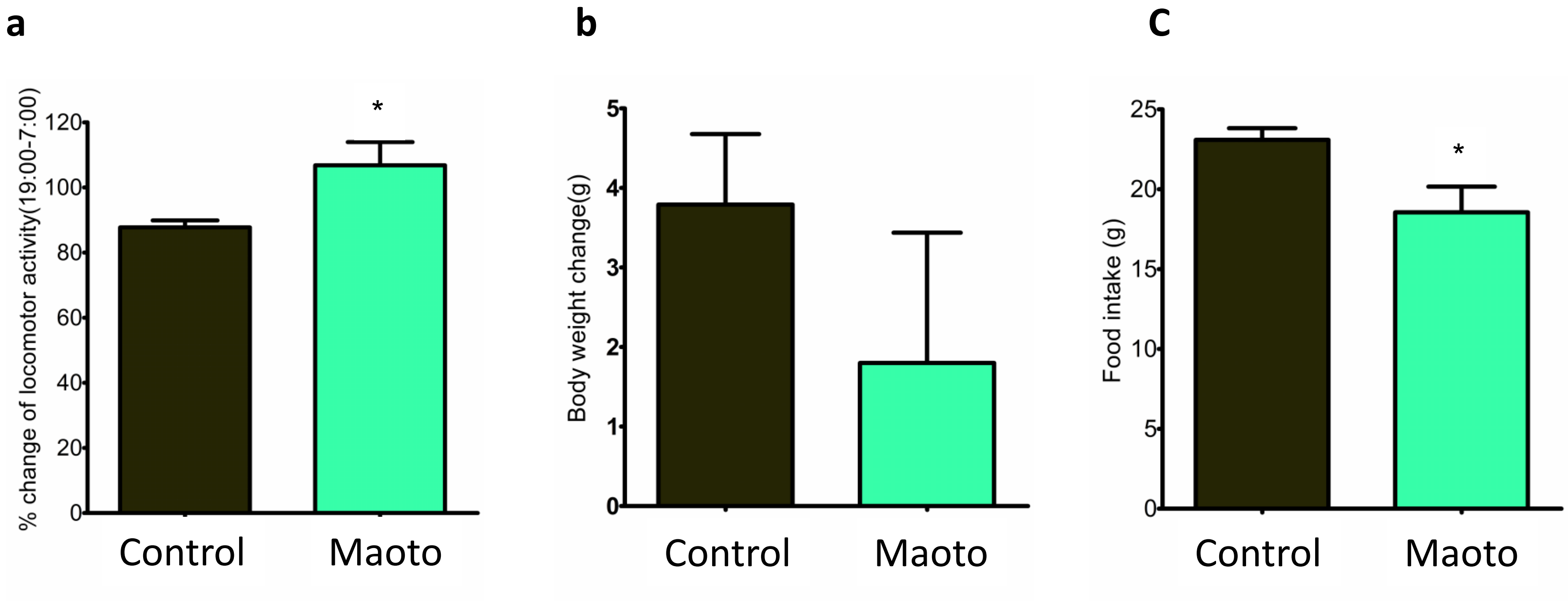

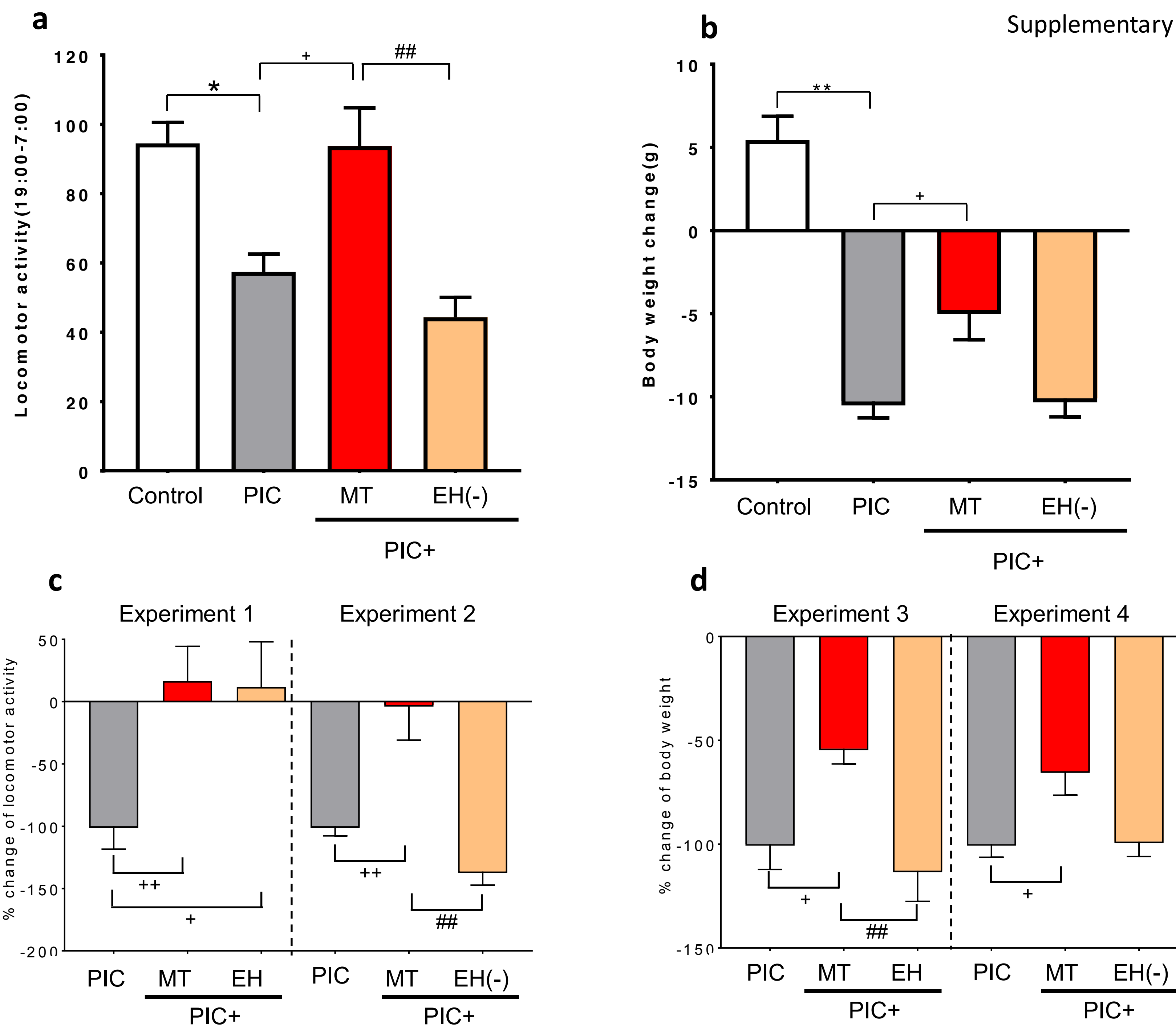
a
b
c
d
Locomotor activity(19:00-7:00)
Body weight change(g)
% change of locomotor activity
% change of body weight
Experiment 1
Experiment 2
Experiment 3
Experiment 4
Control
PIC
MT
EH(-)
EH
PIC+
120
100
80
60
40
20
0
10
5
-5
-10
-15
50
-50
-100
-150
-200
*
**
+
++
##

Supplementary Figure 7

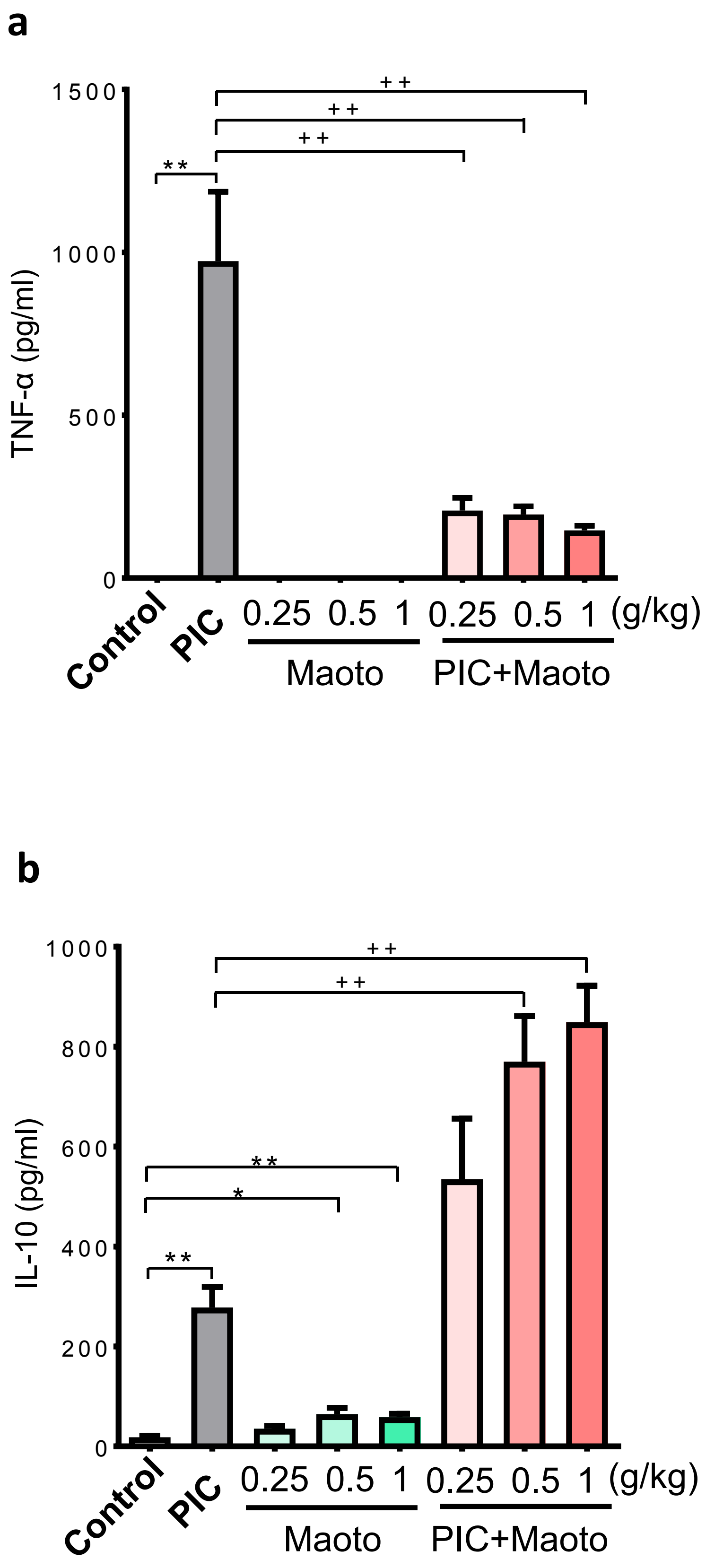

Supplementary Figure 8

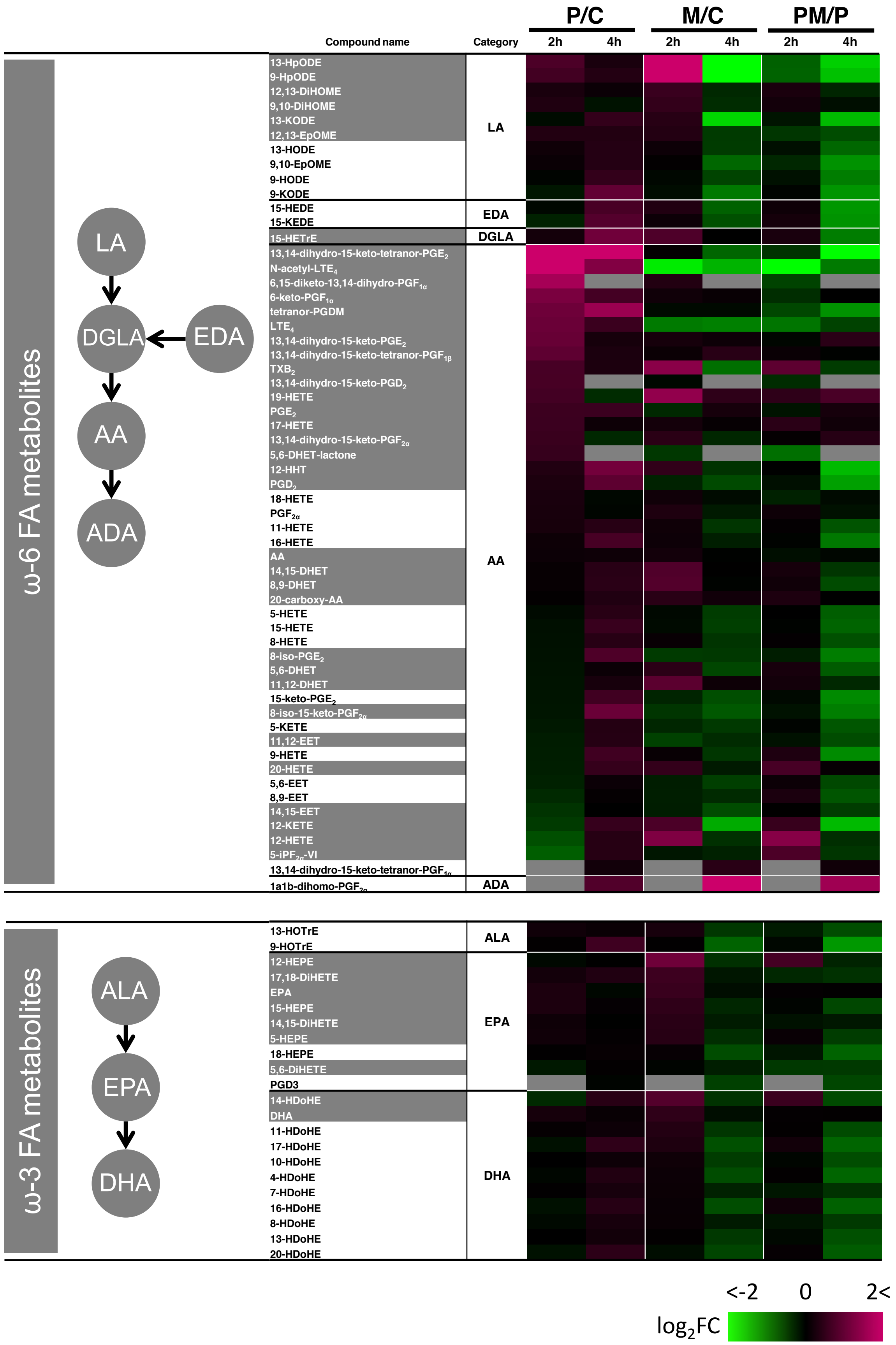

Supplementary information of Figure 6a, b and c

Abbreviation list	
5-iPF2a-VI	5-iso-prostaglandinF2α-VI
6-kPGF1a	6-keto-prostaglandinF1α
6,15-dk-13,14-dhPGF1a	6,15-diketo-13,14-dihydro-prostaglandinF1α
8-iPGE2	8-iso-prostaglandinE2
8-i-15-kPGF2a	8-iso-15-keto-prostaglandinF2α
13,14-dh-15-kPGD2	13,14-dihydro-15-keto-prostaglandinD2
13,14-dh-15-kPGE2	3,14-dihydro-15-keto-prostaglandinE2
13,14-dh-15-ktPGE2	13,14-dihydro-15-keto-tetranor-prostaglandinE2
13,14-dh-15-ktPGF1b	13,14-dihydro-15-keto-tetranor-prostaglandinF1β
13,14-dh-15-ktPGF2a	13,14-dihydro-15-keto-tetranor-prostaglandinF2α
AA	arachidonicacid
cAA	carboxyarachidonicacid
COX	cyclooxygenase
CYP	cytochromeP450
DGLA	dihomo-γ-linolenicacid
DHA	docosahexaenoicacid
DHET	dihydroxy-eicosatrienoicacid
DiHETE	dihydroxy-eicosatetraenoicacid
DiHOME	dihydroxy-octadecenoicacid
EET	epoxy-eicosatrienoicacid
EPA	eicosapentaenoicacid
EpOME	epoxy-octadecenoicacid
HDoHE	hydroxy-docosahexaenoicacid
HETrE	hydroxy-eicosatrienoicacid
HEPE	hydroxy-eicosapentaenoicacid
HETE	hydroxy-eicosatetraenoicacid
HHT	hydroxyheptadecatrienoicacid
HpODE	hydroperoxy-linoleicacid
KETE	oxo-eicosatetraenoicacid
KODE	oxo-octadecadienoicacid
LA	linoleicacid
LOX	lipoxygenase
LTA4	leukotrieneA4
LTC4	leukotrieneC4
LTD4	leukotrieneD4
LTE4	leukotrieneE4
PGD2	prostaglandinD2
PGE2	prostaglandinE2
PGH2	prostaglandinH2
PGI2	prostaglandinI2
tPGDM	tetranor-prostaglandinD metabolite
TXB2	thromboxaneB2

Supplementary information of Figure 6d

Pathway	Metabolites	P/C	M/C	PM/P
COX	6-keto-PGF1α	■		
	6,15-diketo-13,14-dihydro-PGF1α	■		
	11-HETE			
	12-HHT			
	13,14-dihydro-15-keto-PGD2			
	13,14-dihydro-15-keto-PGE2	■		
	13,14-dihydro-15-keto-PGF2α	■		
	13,14-dihydro-15-keto-tetranor-PGE2	■		
	13,14-dihydro-15-keto-tetranor-PGF1β	■		
	15-keto-PGE2			
	PGD2			
	PGE2			
	PGF2α			
	tetranor-PGDM	■		■
	TXB2			
CYP	5,6-DHET		■	
	5,6-DHET-lactone			
	5,6-EET			
	8,9-DHET		■	
	8,9-EET			
	9,10-DiHOME		■	
	9,10-EpOME			
	11,12-DHET			
	11,12-EET		■	
	12,13-DiHOME		■	
	12,13-EpOME			
	14,15-DHET		■	
	14,15-DiHETE		■	
	14,15-EET			
	16-HETE			
	17-HETE			
	17,18-DiHETE		■	
	18-HETE			
	19-HETE		■	
	20-carboxy-AA		■	■
	20-HETE			■
LOX	4-HDoHE			
	5-HEPE		■	
	5-HETE			
	5-KETE			
	5,6-DiHETE			■
	7-HDoHE			
	8-HDoHE			
	8-HETE			
	9-HODE			
	9-HpODE			
	9-KODE			
	10-HDoHE			
	11-HDoHE			
	12-HEPE			
	12-HETE			
	12-KETE			
	13-HDoHE			
	13-HODE			
	13-HpODE			
	13-KODE			
	14-HDoHE			
	15-HEPE		■	
	15-HETE			
	17-HDoHE			
	LTE4	■	■	■
	N-acetyl-LTE4	■		■
		9	13	6

Supplementary information of Figure 6e and f

Proinflammatory lipid mediators
5-HETE
6-keto-PGF1α
6,15-diketo-13,14-dihydro-PGF1α
9-HODE
9,10-DiHOME
9,10-EpOME
12,13-DiHOME
12,13-EpOME
13,14-dihydro-15-keto-PGD2
13,14-dihydro-15-keto-PGE2
15-keto-PGE2
LTE4
PGD2
PGE2
PGF2α

Anti-inflammatory lipid mediators
4-HDoHE
5,6-EET
7-HDoHE
8-HDoHE
8-HETE
10-HDoHE
11,12-DHET
12-HEPE
12-HETE
13-HDoHE
13-HODE
13-HOTrE
14-HDoHE
14,15-DHET
14,15-EET
15-HEPE
15-HETE
15-HETrE
16-HDoHE
16-HETE
17-HDoHE
20-HDoHE
